# National Health Insurance Expenditure and Health-System Use for Hepatitis B and C in Bulgaria, 2021–2025

**DOI:** 10.3390/healthcare14213639

**Published:** 2026-10-27

**Authors:** Kostadin Kostadinov, Stefan Stefanov, Nikola Boyanov, Ani Kevorkyan, Ralitsa Raycheva

**Affiliations:** 1 Department of Social Medicine and Public Health, Faculty of Public Health, Medical University of Plovdiv, 4002 Plovdiv, Bulgaria; kostadinr.kostadinov@mu-plovdiv.bg (K.K.); ralitsa.raycheva@mu-plovdiv.bg (R.R.); 2 Department of Epidemiology and Disaster Medicine, Faculty of Public Health, Medical University of Plovdiv, 4002 Plovdiv, Bulgaria; ani.kevorkyan@mu-plovdiv.bg; 3 Medical Simulation Training Center, Medical Faculty, Medical University of Plovdiv, 4002 Plovdiv, Bulgaria; nikola.boyanov@mu-plovdiv.bg; 4 Department of Gastroenterology, Pulmed University Hospital, 4002 Plovdiv, Bulgaria

**Keywords:** antiviral therapy, Bulgaria, health expenditure, hepatitis B, hepatitis C, reimbursement

## Abstract

**Highlights:**

**What are the main findings?**

- Between 2021 and 2025, the Fisher price component fell by 20.1% for HBV and 11.5% for HCV, while the weighted quantity component rose by 39.5% and 44.7%, respectively.
- Annual antiviral reimbursement increased from EUR 18.9 million to EUR 21.7 million. The share of the NHIF outpatient pharmaceutical budget for all products in the hepatitis case definition, including adjunctive products, declined from 3.99% to 2.61%. Estimated mean continuous HBV therapy increased from 2226 to 3051 people, and HCV standard-course equivalents increased from 604 to 777.

**What are the implications of the main findings?**

- Lower reimbursed unit prices indicate reduced acquisition expenditure per unit of treatment; whether this creates fiscal capacity for treatment expansion requires further assessment.
- Affordability, treatment accessibility, unmet need, and the contribution of diagnosis, referral, and eligibility barriers were not measured.

**Abstract:**

Background: National reimbursement records complement model-based estimates of hepatitis treatment costs. Our primary objective was to quantify Bulgarian National Health Insurance Fund (NHIF) antiviral reimbursement and its price and quantity components; secondary objectives were to estimate dispensing-based treatment volume and describe service activity. Methods: This retrospective study analysed aggregated national pharmacy records for complete years 2021–2025 and hospital and outpatient records for complete years 2024–2025. Diagnosis-identified records were combined with product-identified antiviral prescriptions under fibrosis or cirrhosis codes. Fisher indices decomposed expenditure; seasonal quasi-Poisson models with Newey–West standard errors described monthly trends. Results: Antiviral reimbursement totalled EUR 101.5 million, including EUR 91.6 million for HCV and EUR 10.0 million for HBV. Annual antiviral reimbursement increased from EUR 18.9 million to EUR 21.7 million. The budget share for all pharmacy products in the hepatitis case definition, including adjunctive products, declined from 3.99% to 2.61%. Relative to 2021, the 2025 Fisher price and quantity indices were 0.799 and 1.395 for HBV and 0.885 and 1.447 for HCV. Estimated mean continuous HBV therapy increased from 2226 to 3051 people; HCV standard-course equivalents increased from 604 to 777, with duration-based ranges of 431–607 and 571–777. These estimates do not identify unique treated patients. Annualised inpatient-episode trends during 2024–2025 were 9.2% for HBV and −7.4% for HCV; consultation trends were 3.7% and −6.9%. Conclusions: Dispensed treatment volume increased while reimbursed unit prices and the hepatitis pharmaceutical budget share declined. These findings describe payer expenditure and encounters; they do not establish improved affordability, access, or treatment coverage.

## 1. Introduction

Chronic HBV and HCV infection are major causes of cirrhosis, hepatocellular carcinoma, and premature death [1,2,3]. The World Health Assembly targets for eliminating viral hepatitis as a public health threat by 2030 require substantial gains in diagnosis and treatment coverage [4,5]. Progress remains uneven, including within Europe [6].

Eastern and Southern Europe carry comparatively high rates of HBV- and HCV-related disease [7,8]. Evidence from Bulgaria includes studies in people living with HIV, haemodialysis populations, and people with hepatitis D, but a probability-based national prevalence estimate is not available [9,10,11,12]. A standardised European protocol for general-population HCV surveys was piloted in Bulgaria in response to this evidence gap [13].

Economic evidence on viral hepatitis includes models of lifetime costs, cost effectiveness, and elimination strategies [14,15,16,17,18,19]. For example, a four-country study including Romania modelled future clinical events and cost savings after direct-acting antiviral treatment [15], while an Eastern European and Central Asian analysis evaluated the cost effectiveness of case finding and treatment [18]. Such models address different questions from observed national reimbursement records. Claims analyses can separate changes in actual payer expenditure into price, quantity, and product-availability components [20,21,22]. The question addressed here is how the observed national HBV/HCV reimbursement and its price and quantity components changed in Bulgaria.

Bulgaria is in southeastern Europe, on the western coast of the Black Sea, and had 6,423,207 residents on 31 December 2025 [23,24]. Its compulsory social health insurance system has a single national purchaser, the NHIF, which contracts with healthcare providers; the system does not cover every resident [25]. The NHIF reimburses outpatient antivirals for insured people who meet the relevant treatment and protocol requirements [26,27]. National hepatitis programmes provide a wider policy framework for testing and linkage to care [28,29]. These distinctions matter because national reimbursement records enumerate eligible reimbursed activity rather than all infections, all residents, or all treatment delivered in Bulgaria.

Medicine prices are regulated rather than fixed permanently. The National Council on Prices and Reimbursement of Medicinal Products determines prices and reimbursement values through the Positive Drug List and reference-pricing procedures; subsequent price reviews can reduce reimbursed values [30]. In this study, a product’s observed unit value is the published reimbursed amount divided by dispensed packages. It is not a measure of the patients’ total costs or of net payer expenditure after any rebates not recorded in the source.

The primary objective was to quantify NHIF reimbursement for HBV and HCV antivirals during 2021–2025 and decompose its change into price, quantity, and product entry or exit effects. Secondary objectives were to estimate dispensing-based treatment volume, describe the hepatitis share of the outpatient pharmaceutical budget, and summarise inpatient episodes, treatment-protocol procedures, and outpatient consultations during 2024–2025. We did not estimate infection prevalence, treatment coverage, clinical effectiveness, affordability, or unmet need.

## 2. Materials and Methods

### 2.1. Study Design and Data Sources

We conducted a retrospective descriptive study from the third-party payer perspective. Reporting followed STROBE, RECORD, and RECORD-PE guidance [31,32,33]. The completed reporting checklists are provided in the Supplementary Materials.

Three open, aggregated national NHIF datasets were analysed [34,35,36]. The primary analysis used complete calendar years: January 2021–December 2025 for pharmacy reimbursement (60 months) and January 2024–December 2025 for hospital and outpatient activity (24 months). Pharmacy records report product, regional insurance office, diagnosis, packages, and reimbursement through a ten-day reporting period. Hospital records report monthly activity by facility, pathway or procedure, and diagnosis pair; outpatient records report consultations by practice, specialty, and diagnosis. The population represented comprises all released NHIF records meeting the specified diagnosis and product criteria, rather than a sample of providers. It excludes uninsured care, private expenditure, and records outside those criteria; individual patients cannot be identified or linked across sources.

The available source windows extend from July 2020 to June 2026 for pharmacy reimbursement and January 2024 to July 2026 for hospital and consultation activity. These longer windows are retained only in supplementary descriptive and sensitivity analyses. Pharmacy observations in 2020 and 2026 cover six months each; service observations in 2026 cover seven months. Hospital and consultation observations for 2021–2023 are unavailable and were not reconstructed.

### 2.2. Case Definition

The diagnosis-identified arm used HBV codes B16, B16.0, B16.1, B16.2, B16.9, B17.0, B18.0, and B18.1 and HCV codes B17.1 and B18.2. Non-specific hepatitis codes were considered separately and were not assigned to either virus. The pharmacy drug-identified arm additionally selected antiviral records with K74.0 (hepatic fibrosis) or K74.6 (other and unspecified cirrhosis) and an eligible hepatitis product. HBV assignment used J05AF05, J05AF07, J05AF10, J05AF11, or L03AB11; HCV assignment used the observed J05AP codes, their historical J05AX counterparts, or J05AB04. A list of every observed ATC and NHIF product code is provided in the Supplementary Materials (Table S21). ATC histories were harmonised by retaining each product’s most recent observed ATC code.

Assignment under K74.0 or K74.6 relies on the reimbursed product and its hepatitis prescribing context, supported by the NHIF requirements [26,27], rather than on fibrosis or cirrhosis alone. These codes do not establish viral aetiology, and some included medicines have other indications. Peginterferon alfa-2a can be used for both HBV and HCV; it was assigned to HBV because of the NHIF indication context and its observed diagnosis pattern. This rule was not validated against clinical records. The two arms are mutually exclusive at record level because they contain different diagnosis codes. Sensitivity analyses report diagnosis-identified reimbursement alone and exclude peginterferon from the drug-identified arm (Table S22). Coding changes can redistribute expenditure between arms without representing a change in disease burden.

Hospital records were classified by hepatitis as the primary or secondary diagnosis. Ambulatory procedure 38, used by specialised commissions to issue or renew reimbursed-treatment protocols [26,27], was reported separately from inpatient episodes. Hospital episodes, protocol procedures, and outpatient consultations are encounters: one person may contribute repeatedly within and across services, so these counts cannot be summed to obtain unique patients.

### 2.3. Expenditure and Treatment Volume

Reimbursement was analysed in nominal euro, converting BGN at 1 EUR = 1.95583 BGN. A sensitivity analysis used constant 2025 prices and the Bulgarian all-items harmonised index of consumer prices [37]. Antiviral expenditure comprised observed ATC J05 products and peginterferon alfa-2a (L03AB11). Adjunctive products were separate. The pharmaceutical budget-share numerator was all pharmacy reimbursement within the HBV/HCV study case definition: antivirals from both identification arms plus adjunctive products under the HBV/HCV diagnosis codes. The denominator was all NHIF outpatient pharmaceutical reimbursement in the same calendar year. Consequently, this broader numerator differs from antiviral-only expenditure (Table 1; Figure 1; Table S16).

Package counts were the directly observed volume measure. For oral HBV nucleos(t)ide analogues, patient-month equivalents were calculated as packages × tablets per package ÷ 30.4, assuming one tablet daily. Annual patient-month equivalents divided by the 12 observed months estimated the mean number receiving continuous therapy; this is neither an annual unique-patient count nor an estimate based on peginterferon. HCV direct-acting antiviral packages were converted to standard-course equivalents, using two packs for an eight-week glecaprevir/pibrentasvir course and three packs for a twelve-week course of the other included regimens [27]. Duration-based bounds used two to four packs for glecaprevir/pibrentasvir, three to four for elbasvir/grazoprevir, and two to six for ledipasvir/sofosbuvir. Ribavirin expenditure remained in the antiviral totals, but its packages were not counted as separate HCV courses.

Both conversions assume that dispensed packages correspond to the intended regimen and duration. Interruptions, non-adherence, stockpiling, switching, retreatment, incomplete courses, and dispensing across calendar-year boundaries cannot be resolved. Dispensing may overstate completed therapy, while unobserved or non-NHIF supplies are excluded. HCV estimates are prescriptions converted to course equivalents, not unique initiators, completed courses, or confirmed cures; their duration-based ranges are assumption bounds, not confidence intervals. HBV averages describe ongoing treatment, whereas HCV equivalents describe finite-course dispensing, so their numerical values are not directly comparable patient counts.

### 2.4. Price and Quantity Decomposition

Annual expenditure changes were decomposed with bilateral Fisher indices at NHIF product-code level [21,38,39]. The reference year for fixed-base analyses was 2021 (index = 1). Among products present in both years, Laspeyres uses base-year weights and Paasche comparison-year weights; their geometric mean is the Fisher index. A price index of 0.799 therefore denotes a 20.1% fall in the matched-product price component relative to 2021, rather than a 20.1% fall in total expenditure. Quantity indices describe the corresponding weighted change in packages. An entry-and-exit factor captures the difference between expenditure growth for all products and matched products. The decomposition satisfies:

(1) $$\frac{V_{1}}{V_{0}}=P_{F}\times Q_{F}\times E,$$

where *V*_1_/*V*_0_ is the expenditure ratio, *P_F_* the Fisher price index, *Q_F_* the Fisher quantity index, and *E* is the entry-and-exit factor. The identity was tested on synthetic panels and verified for every comparison. Fixed-base analyses compared 2022 through 2025 with 2021; chained analyses compared consecutive years. Alternative price indices and molecule-level decompositions are reported in the Supplementary Materials.

The factors are multiplicative: percentage changes in price, quantity, and entry or exit must not be added. Indices below 1 indicate a decrease and above 1 an increase relative to the reference year. Product turnover can also reflect changes in reimbursed product codes or availability, so the residual is not interpreted as a causal effect of market entry.

### 2.5. Health-System Activity and Statistical Analysis

Annual hospital episodes, treatment-protocol procedures, and outpatient consultations were summarised by virus. Hospital activity was also valued at 2025 National Framework Contract tariffs as a volume-based estimate rather than an observed payment total [40].

Monthly trends were estimated with log-link quasi-Poisson models containing linear time and one annual sine/cosine pair. This parsimonious specification estimates a smooth descriptive trend while allowing seasonal variation and overdispersion; it cannot establish causality. The primary windows provided 60 expenditure observations and 24 service observations. Newey–West covariance used Bartlett weights, lag 3, no prewhitening, and no finite-sample adjustment; confidence intervals and two-sided *p*-values used normal Wald inference. Annualised changes were calculated as [exp(12β) − 1] × 100. Sensitivity analyses used the full source windows, a model without seasonal terms, and covariance lags 0, 1, and 6; log-linear expenditure models provided an additional check (Table S20). No model resolved the limited service follow-up of two complete annual cycles. The January 2026 interruption analysis remains supplementary and exploratory. Analyses used R version 4.6.1 (R Foundation for Statistical Computing, Vienna, Austria); revision analyses used an explicit Bartlett sandwich implementation checked against the reported full-window estimates.

### 2.6. Ethics

Ethical approval was not required because all sources were public, aggregated datasets without personal data.

## 3. Results

### 3.1. Records and Reimbursed Expenditure

Annual antiviral reimbursement increased from EUR 18.9 million in 2021 to EUR 21.7 million in 2025. Separately, reimbursement for all pharmacy products within the HBV/HCV case definition accounted for 3.99% and 2.61% of the total NHIF outpatient pharmaceutical reimbursement, respectively (Table 1; Figure 1). In the complete-year seasonal models, annualised antiviral reimbursement trends were 1.4% for HBV and 5.4% for HCV. Log-linear sensitivity estimates were 1.2% and 5.7%; full-window seasonal estimates were 1.7% and 2.2% (Table S20). The HCV trend magnitude therefore depends on the analysis window.

The budget-share numerator includes antivirals from both case-definition arms and adjunctive products under HBV/HCV diagnoses; it is broader than the antiviral-only amounts in the expenditure columns. All observations in this table cover complete calendar years.

The complete-year analysis included 23,740 pharmacy records across 60 months, 16,716 hospital records, and 35,009 consultation records across 24 months. Antiviral reimbursement totalled EUR 101.5 million: EUR 91.6 million for HCV and EUR 10.0 million for HBV. The drug-identified arm accounted for 20.6% of this total, comprising 19.0% for HCV and 1.5% for HBV (Table 2; component shares may not sum after rounding). The full-window counts and expenditures are retained in the Supplementary Materials.

### 3.2. Price, Quantity, and Treatment Volume

Relative to 2021, the 2025 HBV expenditure ratio was 1.083, equal to a Fisher price index of 0.799 × quantity index of 1.395 × entry-and-exit factor of 0.971. Thus, a 20.1% lower price component and 39.5% higher quantity component coexisted with an 8.3% increase in total reimbursement. For HCV, 0.885 × 1.447 × 0.903 = 1.157: the price component fell 11.5%, the quantity component rose 44.7%, and total reimbursement rose 15.7% (Table 3; Figure 2). These products of rounded indices are approximate; the identity holds using unrounded values. The entry-and-exit factors were 2.9% and 9.7% below 1, respectively. Constant-2025-price reimbursement declined from EUR 2.66 million to EUR 2.21 million for HBV and EUR 22.0 million to EUR 19.5 million for HCV (Table S8).

Estimated mean continuous HBV therapy increased from 2226 in 2021 to 3051 in 2025. HCV standard-course equivalents increased from 604 to 777, with duration-based ranges of 431–607 and 571–777, respectively. Mean reimbursed direct-acting antiviral expenditure per HCV standard-course equivalent declined from EUR 27,897 to EUR 25,126 (Figure 3; Table S5). During complete years 2024–2025, treatment-protocol procedures averaged 5345 per year for HBV and 696 for HCV. These encounters are reported separately and do not validate unique treated-patient counts; full-window protocol counts are shown in Table S14.

### 3.3. Hospital and Outpatient Activity

In 2024 and 2025, primary-diagnosis inpatient episodes increased from 6504 to 7020 for HBV and decreased from 1496 to 1401 for HCV. Treatment-protocol procedures increased from 5044 to 5646 for HBV and decreased from 708 to 684 for HCV. Outpatient consultations increased from 17,322 to 18,114 for HBV and decreased from 4491 to 4278 for HCV (Table 4).

All observations in Table 4 cover complete calendar years. The Supplementary Materials retain partial-year 2026 observations and separately report episodes with hepatitis as a secondary diagnosis (Table S6).

During 2024–2025, annualised inpatient-episode trends were 9.2% for HBV and −7.4% for HCV. Protocol-procedure trends were 12.7% and −5.0%, and consultation trends were 3.7% and −6.9%, respectively (Table 5; Figure 4). The HCV protocol-procedure interval included no change. Directions were unchanged when seasonal terms were removed or the full 31-month service window was used; magnitudes and confidence intervals varied (Table S20). These are descriptive encounter trends over a short period.

Valued at 2025 tariffs, hospital activity totalled EUR 6.04 million in 2025. This comprised EUR 4.88 million for HBV inpatient episodes, EUR 0.96 million for HCV inpatient episodes, and EUR 0.19 million for treatment-protocol procedures (Table S12).

## 4. Discussion

### 4.1. Principal Findings

Between complete years 2021 and 2025, national antiviral reimbursement increased while weighted quantities rose and reimbursed unit prices fell. The broader hepatitis-related share of the outpatient pharmaceutical budget also declined. Together, these measures show increased dispensing without a proportional increase in reimbursement; they do not demonstrate improved treatment access, affordability, or reduced unmet need.

The treatment-volume measures complement expenditure but answer different questions. HBV estimates describe the mean volume of ongoing daily oral therapy, while HCV estimates describe finite-course equivalents inferred from dispensing. Interruptions, regimen duration, repeat treatment, and incomplete courses affect their interpretation. Protocol procedures provide a separate activity series within the same reimbursement system; repeated approvals and different time units prevent their use as independent validation of the package-based estimates.

These trends are consistent with the different treatment trajectories of the two infections. The decline observed for HCV may partly reflect finite treatment followed by cure, whereas increasing HBV activity is consistent with the accumulation of patients requiring long-term suppressive therapy. The datasets do not link diagnosis, treatment, cure, and subsequent service use within individuals, so changes in coding, referral, clinical practice, or the pool of diagnosed people could also contribute.

### 4.2. Comparison with Other Evidence

The decline in reimbursed prices is consistent with claims analyses showing lower HCV medicine prices after competition among direct-acting antiviral regimens and lower HBV nucleos(t)ide analogue expenditure after generic entry [20,21,22]. The explicit entry-and-exit factor used here addresses a limitation of simple average-price comparisons when the reimbursed product set changes. In the Bulgarian data, declining price indices coexisted with rising quantity indices for both viruses.

The Bulgarian price-setting system permits reimbursement values to change following reference-price reviews [30]. The observed 2021–2025 price indices are consistent with lower reimbursed unit values, but the analysis cannot attribute those changes specifically to regulation, competition, negotiated discounts, or generic entry. Published claim amounts also need not capture any subsequent rebates. Accordingly, the indices describe the recorded price component rather than an evaluation of a particular pricing policy.

The absolute treatment volumes cannot be converted into national coverage because a representative prevalence denominator is unavailable. Modelled prevalence and burden estimates provide context but are not sufficiently precise substitutes for a national probability sample [7,41]. Reporting a coverage percentage from those sources would combine uncertainty in the numerator, particularly for HCV course duration, with uncertainty in the denominator.

### 4.3. Implications

Lower reimbursed unit prices may create fiscal capacity to supply more treatment, but fiscal capacity was not directly estimated. The falling budget share also reflects growth in the overall outpatient pharmaceutical denominator. Neither indicator establishes patient affordability, insurance coverage, geographical access, timely initiation, or the amount of unmet need. Diagnosis, referral, treatment eligibility, and acceptance are plausible areas for further investigation rather than barriers identified by this analysis.

Bulgaria’s national hepatitis programme for 2026–2030 adds testing settings and mechanisms, including rapid testing through public health services, outpatient facilities, laboratories, and non-governmental organisations [29]. Administrative linkage across testing, referral, protocol approval, dispensing, and clinical outcomes would be needed to determine where patients are lost from the pathway. Such data would also allow treatment volume to be estimated directly rather than inferred from packages.

The national HBV reimbursement requirements also merit evaluation against current clinical guidance. The NHIF criteria combine virological, biochemical, and fibrosis measures [26], while recent European and American guidance supports treatment in some patients at lower biochemical thresholds [42,43]. The present study cannot estimate how many people are affected by that difference. A patient-level eligibility audit would be required before its effect on access or expenditure could be quantified.

### 4.4. Strengths and Limitations

The study measures national reimbursed expenditure rather than projecting costs from a model. The analysis separates price, quantity, and product turnover; verifies the decomposition identity; and tests monetary trends with an alternative model. The case definition also recovers antiviral prescriptions recorded under fibrosis or cirrhosis diagnoses that would be missed by hepatitis codes alone.

The aggregated design prevents the identification of unique patients, adherence, completion, cure, clinical severity, or transitions between services. The selected NHIF records do not cover uninsured or privately financed care and cannot establish that every treated person is captured. Package-based treatment estimates depend on regimen and dispensing assumptions. Drug-based assignment under fibrosis or cirrhosis codes was not clinically validated; medicines with shared indications introduce potential misclassification. Hospital tariffs value activity rather than observed payments, and expenditure outside the recorded NHIF reimbursement is excluded. Administrative coding changes can alter recorded composition. Complete-year service models contain only 24 months; the full-window sensitivity has 31 months, and the supplementary interruption analysis only six post-interruption observations. Seasonal specification, covariance lag, and endpoint choice affect estimates. All temporal models are descriptive and cannot identify effects of policy, pricing mechanisms, or treatment access.

## 5. Conclusions

During complete years 2021–2025, NHIF records in Bulgaria showed increasing dispensed HBV and HCV treatment volume, falling reimbursed unit prices, and a declining share of the outpatient pharmaceutical budget for all products in the HBV/HCV case definition. Service activity during 2024–2025 showed rising HBV and declining HCV encounter trends. Lower unit prices could support future expansion, but these data do not measure affordability, access, unmet need, or treatment coverage. Evaluating those outcomes requires linked patient-level data and an appropriate population denominator.

## Figures and Tables

**Figure 1:**
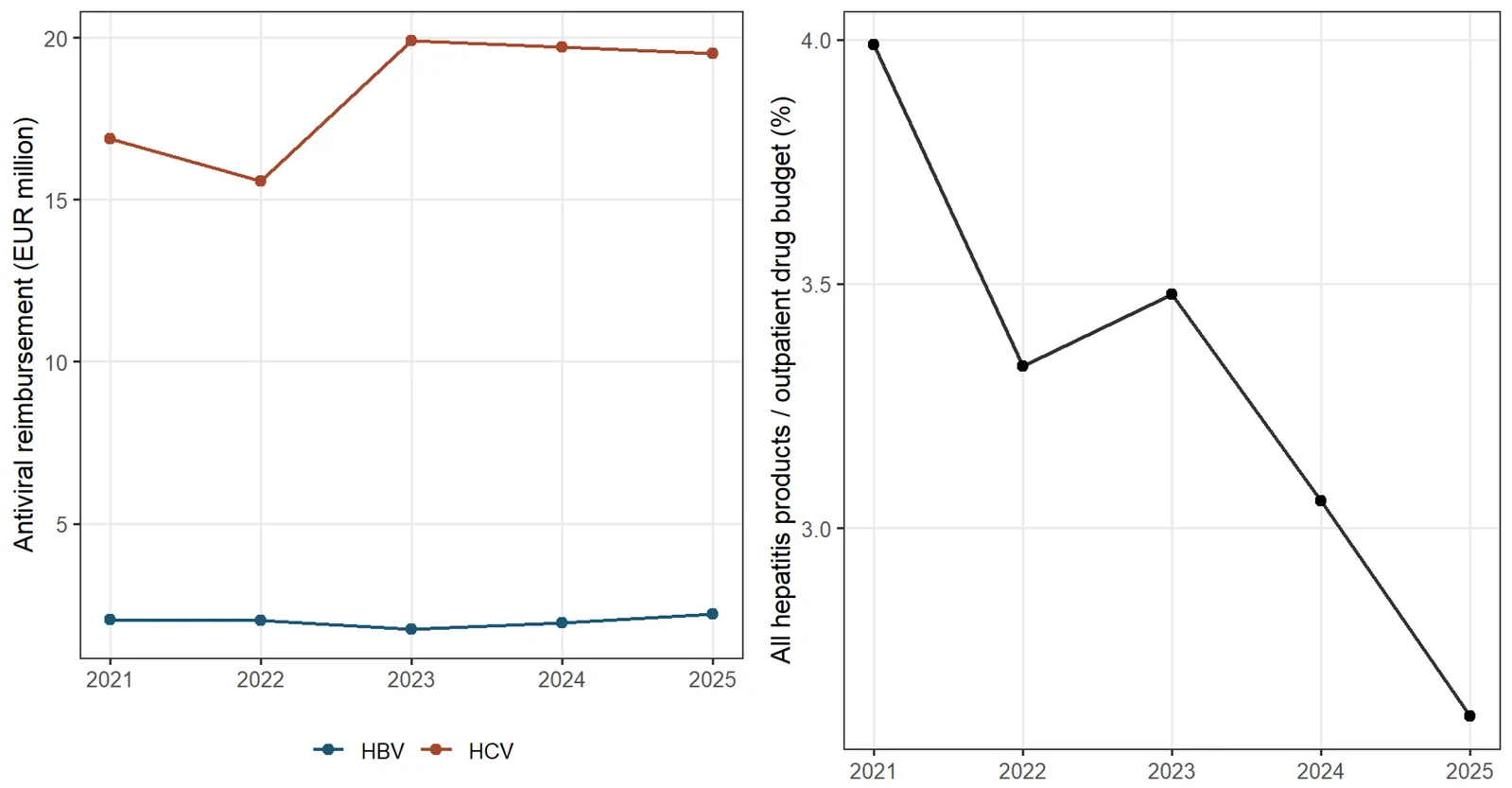
Complete-year antiviral reimbursement in 2021–2025 (**left**) and the separate budget share for all pharmacy products in the HBV/HCV case definition (**right**). The budget-share numerator includes antivirals from both identification arms and adjunctive products; its denominator is all NHIF outpatient pharmaceutical reimbursement in the same year.

**Figure 2:**
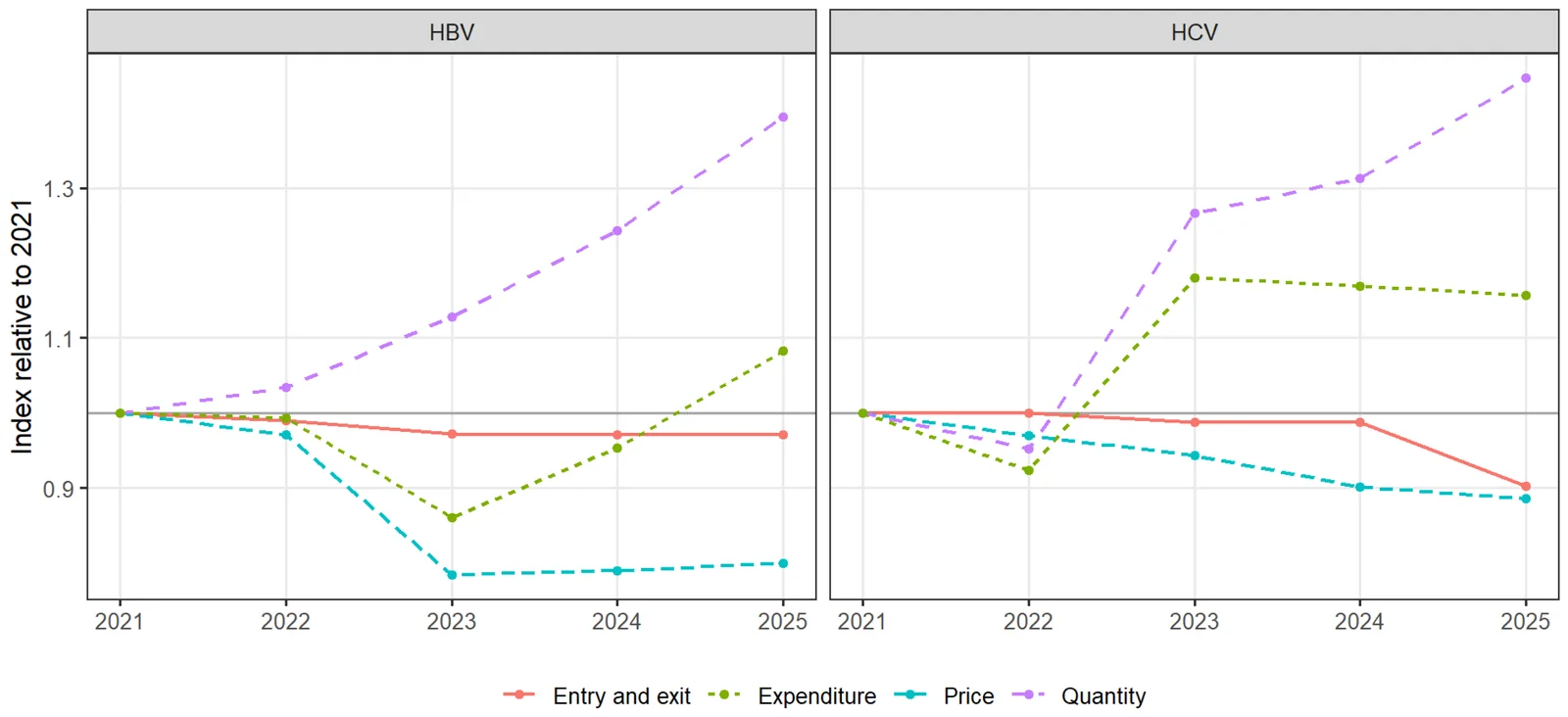
Fisher decomposition of antiviral reimbursement during complete years 2021–2025. Price, quantity, product entry and exit, and total-expenditure indices use 2021 = 1. The three component factors multiply to the total-expenditure ratio. The grey horizontal line marks an index of 1 (no change from 2021).

**Figure 3:**
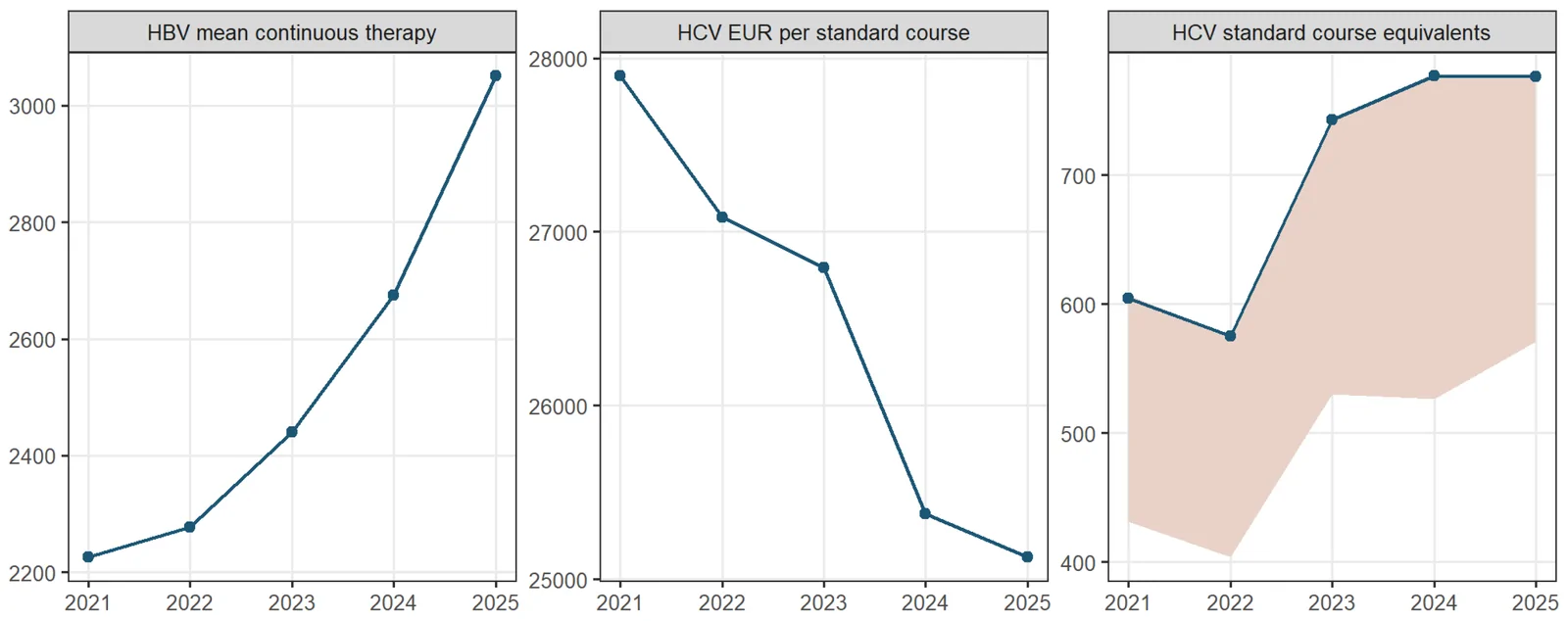
Dispensing-based treatment estimates and HCV reimbursed direct-acting antiviral expenditure per standard course, 2021–2025. The HCV shaded band reflects alternative permitted durations rather than statistical uncertainty. HBV values are annual means of continuous-therapy equivalents; HCV values are finite-course equivalents. Neither measure counts unique patients.

**Figure 4:**
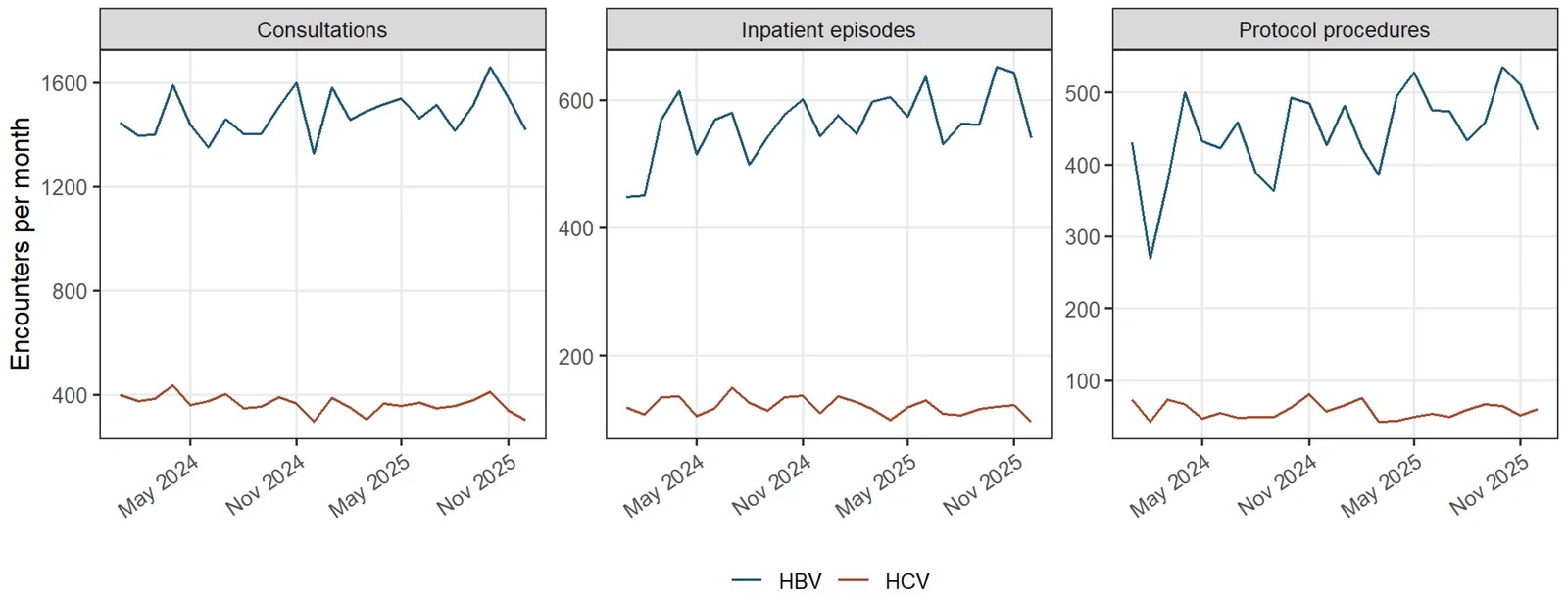
Monthly NHIF encounters during January 2024–December 2025. Inpatient episodes, treatment-protocol procedures, and outpatient consultations are separate activity measures and do not identify unique patients.

**Table 1:** Annual NHIF antiviral reimbursement and dispensing-based treatment estimates during complete years 2021–2025, in nominal euro. The budget share uses the broader all-products hepatitis numerator defined in the Methods section, and the note below; it is not an antiviral-only share.

Year	Hepatitis B, EUR	Hepatitis B Mean Continuous Therapy Equivalents	Hepatitis C, EUR	Hepatitis C, Standard-Course Equivalents (Range)	All Hepatitis Products Share of NHIF Outpatient Drug Budget, %
2021	2,037,503	2226	16,871,845	604 (431 to 607)	3.99
2022	2,022,898	2277	15,572,863	575 (404 to 575)	3.33
2023	1,752,227	2441	19,902,588	743 (530 to 743)	3.48
2024	1,941,433	2675	19,714,761	777 (526 to 777)	3.06
2025	2,205,974	3051	19,514,347	777 (571 to 777)	2.61

**Table 2:** Antiviral reimbursement by mutually exclusive case-definition arm during complete years 2021–2025. Diagnosis-identified records use HBV/HCV diagnosis codes; drug-identified records use K74.0 or K74.6 plus the product assignment rules. Percentages use all HBV/HCV antiviral reimbursement in this period as the denominator. Drug-based assignment is an administrative assumption, not clinical validation.

Arm of the Case Definition	Virus	Records	Reimbursement, EUR	Share of Antiviral Spend, %
Diagnosis-identified	Hepatitis B	8501	8,390,788	8.3
Diagnosis-identified	Hepatitis C	2203	72,254,753	71.2
Drug-identified (liver-disease code)	Hepatitis B	6329	1,569,247	1.5
Drug-identified (liver-disease code)	Hepatitis C	1089	19,321,652	19.0

**Table 3:** Product-level Fisher decomposition of antiviral reimbursement from 2021 to 2025. Price, quantity, and entry/exit factors multiply to the expenditure ratio, exactly before rounding. The reference is 2021 = 1. Intermediate fixed-base and chained comparisons are retained in Table S23; molecule-level alternative indices are presented in Table S3.

Virus	2021 EUR	2025 EUR	Expenditure Ratio	Price Index	Quantity Index	Entry and Exit	Matched Products
HBV	2,037,503	2,205,974	1.083	0.799	1.395	0.971	7
HCV	16,871,845	19,514,347	1.157	0.885	1.447	0.903	3

**Table 4:** NHIF inpatient episodes, treatment-protocol procedures, and outpatient consultations for HBV/HCV during complete years 2024–2025. These are encounters, not unique patients. Protocol procedures are ambulatory procedure 38 and are separate from inpatient episodes.

Year	Inpatient, Hep. B	Inpatient, Hep. C	Protocol, Hep. B	Protocol, Hep. C	Consultations, Hep. B	Consultations, Hep. C
2024	6504	1496	5044	708	17,322	4491
2025	7020	1401	5646	684	18,114	4278

**Table 5:** Descriptive monthly trends in complete-year data: reimbursement in 2021–2025 (60 months), and service activity in 2024–2025 (24 months). Quasi-Poisson models include linear time and annual sine/cosine terms; 95% confidence intervals and *p*-values use normal Wald inference with Bartlett Newey–West covariance at lag 3. Annualised changes are model-derived, not observed year-on-year percentages.

Series	Months	Monthly Ratio (95% CI)	Annualised Change, %	*p*
Antiviral reimbursement, hepatitis B	60	1.0011 (0.9993 to 1.0030)	+1.4	0.219
Antiviral reimbursement, hepatitis C	60	1.0044 (1.0025 to 1.0063)	+5.4	<0.001
Inpatient episodes, hepatitis B	24	1.0074 (1.0030 to 1.0118)	+9.2	<0.001
Inpatient episodes, hepatitis C	24	0.9936 (0.9898 to 0.9974)	−7.4	<0.001
Treatment-protocol procedures, hepatitis B	24	1.0100 (1.0053 to 1.0147)	+12.7	<0.001
Treatment-protocol procedures, hepatitis C	24	0.9958 (0.9889 to 1.0026)	−5.0	0.225
Outpatient consultations, hepatitis B	24	1.0030 (1.0012 to 1.0048)	+3.7	<0.001
Outpatient consultations, hepatitis C	24	0.9941 (0.9904 to 0.9978)	−6.9	0.002

## Data Availability

The source datasets are available on Zenodo under CC BY 4.0: outpatient pharmacy reimbursement, https://doi.org/10.5281/zenodo.22179559 [34]; hospital activity, https://doi.org/10.5281/zenodo.22179552 [35]; and outpatient consultations, https://doi.org/10.5281/zenodo.22179578 [36]. Population denominators and price indices are from the National Statistical Institute and Eurostat [23,37]. The analysis code reproduces the tables and figures from the source files.

## Supplementary Materials

The following supporting information can be downloaded at: https://www.mdpi.com/article/10.3390/healthcare14213639/s1, Table S1: Distribution of expenditure, hospital admissions and outpatient consultations across the ICD-10 case definition; Table S2: Every antiviral product in the HBV/HCV case definition, including diagnosis- and drug-identified arms, July 2020–June 2026; Table S3: Fisher decomposition at molecule (ATC) level with alternative price indices; Table S4: Herfindahl-Hirschman index of reimbursement concentration, computed on cost shares at product and molecule level; Table S5: HCV standard-course equivalents and direct-acting antiviral reimbursement per standard course, by regimen; Table S6: Hospital admissions in which hepatitis B or C was recorded as the secondary diagnosis, by primary discharge diagnosis, for diagnoses with at least 20 admissions; Table S7: Clinical pathways, ambulatory procedures and clinical procedures under which hepatitis admissions were billed, for codes accounting for at least 0.3% of admissions; Table S8: Nominal and constant-2025-price antiviral reimbursement, deflated by the Bulgarian all-items harmonised index of consumer prices; Table S9: Adjunctive products under HBV/HCV diagnoses in the full pharmacy window, July 2020–June 2026; Table S10: Antiviral reimbursement by regional health insurance office, 2021 to 2025, in absolute terms and per 100,000 person-years; Table S11: Interrupted time series at January 2026, when Bulgaria adopted the euro and, the following month, the NHIF revised its treatment requirements; Table S12: Hepatitis hospital activity valued at the 2025 National Framework Contract tariff schedule; Table S13: The 2025 tariffs applied, by pathway and procedure code; Table S14: Treatment-protocol procedures in the full January 2024–July 2026 source window; Table S15: National Health Insurance Fund data sources analysed, their coverage and the number of records meeting the hepatitis case definition; Table S16: Full-window annual antiviral reimbursement and dispensing-based treatment estimates, July 2020–June 2026; Table S17: Log-linear monthly monetary-expenditure sensitivity models, with annual sine/cosine terms and Bartlett Newey–West covariance at lag 3; Figure S1: Annual composition of antiviral reimbursement by leading product; Figure S2: Nominal and constant-2025-price antiviral reimbursement, 2021 to 2025; Figure S3: Herfindahl-Hirschman index of reimbursement concentration by virus and year, at product and molecule level; Figure S4: Antiviral reimbursement per 100,000 person-years by processing regional health insurance office, 2021 to 2025; Figure S5: Annual reimbursed cost per package for the leading antiviral molecules; Table S18: STROBE checklist; Table S19: RECORD checklist; Table S20A: Complete-year and full-window seasonal quasi-Poisson models; Table S20B: Complete-year sensitivity to seasonal specification and covariance lag; Table S21: Observed antiviral identification codes; Table S22: Case-definition sensitivity during complete years 2021–2025; Table S23: Product-level Fisher decomposition for all complete-year comparisons.

## Abbreviations

The following abbreviations are used in this manuscript:

NHIF	National Health Insurance Fund
HBV	Hepatitis B virus
HCV	Hepatitis C virus
