# Supplementary material for: National Health Insurance Expenditure and Health-System Use for Hepatitis B and C in Bulgaria, 2021–2025
Source: Healthcare (Basel). 2026 Oct 27;14(21):3639. doi: 10.3390/healthcare14213639 (PMC13762574; doi:10.3390/healthcare14213639)
Supplement: Supplementary file 1 [file healthcare-14-03639-s001.zip › healthcare-4597029-supplementary.pdf]

# Supplementary material

National Health Insurance Expenditure and Health-System Use for Hepatitis B and C in Bulgaria, 2021–2025

Primary analyses use complete years 2021–2025 for reimbursement and 2024–2025 for service activity. Tables S1–S16 and Figures S1–S5 retain the source windows stated in their captions for descriptive context; their partial-year totals are not compared with complete-year totals. Pharmacy data for 2020 and 2026 cover July–December and January–June, respectively. Service data for 2026 cover January–July. Table S17 and the additional Tables S20–S23 distinguish primary analyses from full-window analyses.

## Supplementary methods

### Case definition in full

Records were extracted on the ICD-10 code as published in the source datasets and assigned to a virus group as follows.

| Group | ICD-10 codes | Treatment in the analysis |
|---|---|---|
| Hepatitis B | B16, B16.0, B16.1, B16.2, B16.9, B17.0, B18.0, B18.1 | Reported as hepatitis B |
| Hepatitis C | B17.1, B18.2 | Reported as hepatitis C |
| Unspecified viral hepatitis | B18, B18.8, B18.9, B19, B19.0, B19.9, Z22.5 | Reported separately; never attributed to a virus |
| Other viral hepatitis | B15, B15.0, B15.9, B17, B17.2, B17.8, B17.9 | Extracted for contrast only; hepatitis A and E |

*ICD-10 case definition. B19.1 and B19.2, which appear in some published hepatitis code lists, are ICD-10-CM codes with no counterpart in the WHO ICD-10 that the Bulgarian classification follows, and do not occur in these data.*

Stage was assigned from the code itself: B15, B16 and B17 are acute, B18 is chronic, B19 is unspecified and Z22.5 is carriage.

The drug-identified arm selects K74.0 (hepatic fibrosis) or K74.6 (other and unspecified cirrhosis of liver) together with an eligible hepatitis antiviral product. Fibrosis or cirrhosis alone is insufficient for viral assignment. The NHIF hepatitis treatment requirements support the prescribing context [26,27], but product-based assignment remains an administrative algorithm

without validation against clinical records. Some medicines have indications beyond a single hepatitis virus. Record-level overlap between the arms is prevented by their disjoint diagnosis-code definitions. Administrative shifts between hepatitis and liver-disease coding can change the arm distribution without a true change in infection burden.

| **Assigned to** | **ATC codes** | **Products** |
|---|---|---|
| Hepatitis B | J05AF05, J05AF07, J05AF10, J05AF11, L03AB11 | lamivudine, tenofovir disoproxil, entecavir, telbivudine, peginterferon alfa-2a; assignment follows the algorithm and prescribing context, not exclusive molecular indications |
| Hepatitis C | J05AP*, J05AX6*, J05AB04 | the direct-acting antiviral regimens and ribavirin |

*Assignment of virus from the prescribed product in the drug-identified arm.*

Peginterferon alfa-2a has historically been used for HBV and HCV. It is assigned to HBV here on the basis of the NHIF prescribing context and its observed diagnosis pattern; no peginterferon record carries an HCV diagnosis in the supplied extract. This assumption does not demonstrate virus specificity. Reimbursement in its drug-identified arm was EUR 25,742 in the full window and EUR 21,219 during 2021–2025. Table S22 shows the effect of excluding these records, alongside a diagnosis-only definition.

Medicines were classified as antiviral where the ATC code begins J05 or equals L03AB11 (peginterferon alfa-2a), and adjunctive otherwise. Adjunctive products reimbursed under a hepatitis diagnosis were silymarin (A05BA03), ademetionine (A16AA02) and ornithine-aspartate (A05BA).

## Treatment course definitions

For oral HBV nucleos(t)ide analogues, dispensed patient-month equivalents equal packages × tablets per package ÷ 30.4, assuming one tablet daily. Dividing by the number of observed months gives the mean continuous-therapy equivalent; the complete-year analysis divides by 12. Peginterferon is not included in this conversion. For HCV, direct-acting antiviral packages are divided by the regimen-specific package requirements shown below. Ribavirin packages are not counted as separate HCV courses, although their reimbursement remains in antiviral expenditure. These measures cannot identify unique patients, treatment initiation, completion, or cure.

| Regimen | ATC | Standard duration | Permitted duration | Standard packages | Sensitivity range |
|---|---|---:|---:|---:|---:|
| glecaprevir/pibrentasvir | J05AP57 | 8 weeks | 8 to 16 weeks | 2 | 2 to 4 |
| sofosbuvir/velpatasvir | J05AP55 (formerly J05AX69) | 12 weeks | 12 weeks | 3 | 3 |
| elbasvir/grazoprevir | J05AP54 (formerly J05AX68) | 12 weeks | 12 to 16 weeks | 3 | 3 to 4 |
| sofosbuvir/velpatasvir/voxilaprevir | J05AP56 | 12 weeks | 12 weeks | 3 | 3 |
| ledipasvir/sofosbuvir | J05AP51 (formerly J05AX65) | 12 weeks | 8 to 24 weeks | 3 | 2 to 6 |

*Definitions used to convert dispensed packages into standard-course equivalents. The sensitivity range incorporates permitted regimen durations and does not represent statistical uncertainty. The resulting values are neither unique-patient counts nor confirmed cures.*

Treatment interruptions, non-adherence, stockpiling, switches, retreatment, incomplete courses, and dispensing across year boundaries are unobservable. The duration range addresses alternative regimen lengths only, and is not a confidence interval or a measure of all uncertainty. Dispensing can overstate completed therapy; supplies outside NHIF reimbursement are excluded. The HBV mean describes ongoing treatment, whereas HCV course equivalents describe finite-course dispensing.

## Fisher decomposition

For a pair of years 0 and 1, let $p_i$ and $q_i$ be the unit value and quantity of product $i$, and let $M$ be the set of products present in both years. The Laspeyres and Paasche price indices on the matched sample are

$$P_L = \frac{\sum_{i \in M} p_{i1}\, q_{i0}}{\sum_{i \in M} p_{i0}\, q_{i0}}, \qquad P_P = \frac{\sum_{i \in M} p_{i1}\, q_{i1}}{\sum_{i \in M} p_{i0}\, q_{i1}} \qquad \text{(S1)}$$

and the Fisher price index is their geometric mean, $P_F = \sqrt{P_L P_P}$; the quantity indices $Q_L$, $Q_P$ and $Q_F$ are defined symmetrically. Writing $V$ for total expenditure and $V^M$ for expenditure on the matched sample, the entry-and-exit factor is

$$E = \frac{V_1/V_0}{V_1^M/V_0^M} \qquad \text{(S2)}$$

so that $V_1/V_0 = P_F \times Q_F \times E$ exactly. The implementation was checked on synthetic panels before use: a panel in which one product's price doubles with quantity held constant must return $P_F = 2$, $Q_F = 1$ and $E = 1$, and a panel with an entrant in the comparison year must satisfy the multiplicative identity to machine precision. Package size was verified constant within every NHIF product code, so a change in unit value is a change in price and not a change in pack size.

## Data quality

**ATC reclassification.** The WHO moved the direct-acting antivirals from J05AX to J05AP during 2024, so three NHIF product codes carry one ATC code before the change and another after it while remaining the same product. Each product code takes the most recent ATC code observed for it, so molecule-level indices do not read the reclassification as simultaneous exit and entry. This is a property of the classification, not a deposit defect, and the handling remains necessary.

**Residual quality.** Within the hepatitis subset there are no negative reimbursement amounts, no zero-package records and no diagnosis code that fails to classify, and package size is constant within every NHIF product code — which is what makes the unit-value panel behind the Fisher decomposition legitimate. One product code carries a missing package size and contributes EUR 499 across seven records; it is excluded from the people-on-therapy conversion and retained elsewhere.

# Supplementary tables

*Table S1. Distribution of expenditure, hospital admissions and outpatient consultations across the ICD-10 case definition. Codes that do not identify a virus are reported here and are never attributed to hepatitis B or C in the main analysis.*

| Source | Virus group | Stage | Value | Share of source, % |
|---|---|---|---|---|
| Hospitalisations, primary diagnosis (cases) | HBV | Acute | 399 | 1.0 |
| | HBV | Chronic | 31,870 | 78.3 |
| | HCV | Acute | 106 | 0.3 |
| | HCV | Chronic | 5,319 | 13.1 |
| | Other viral hepatitis | Acute | 1,741 | 4.3 |
| | Unspecified | Chronic | 60 | 0.1 |
| | Unspecified | Unspecified | 1,231 | 3.0 |
| Outpatient consultations (visits) | HBV | Acute | 995 | 1.5 |

| Source | Virus group | Stage | Value | Share of source, % |
|---|---|---|---|---|
| | HBV | Chronic | 45,237 | 66.6 |
| | HCV | Acute | 323 | 0.5 |
| | HCV | Chronic | 10,819 | 15.9 |
| | Other viral hepatitis | Acute | 1,983 | 2.9 |
| | Unspecified | Carriage | 2,014 | 3.0 |
| | Unspecified | Chronic | 4,923 | 7.2 |
| | Unspecified | Unspecified | 1,658 | 2.4 |
| Pharmacy expenditure (EUR) | HBV | Chronic | 12,507,255 | 10.3 |
| | HCV | Chronic | 109,378,661 | 89.7 |

*Table S2. Every antiviral product in the HBV/HCV case definition, including diagnosis- and drug-identified arms, July 2020–June 2026. This full-window inventory is descriptive. Product absence for the final six months is labelled withdrawn from the observed series, without implying formal withdrawal of marketing authorisation. Exact NHIF codes and historical ATC mappings are in Table S21.*

| Virus | Active substance | ATC | Brand | Strength | Pack size | First month | Last month | Packages | Reimbursement, EUR | Cost per package, EUR | Status |
|---|---|---|---|---|---|---|---|---|---|---|---|
| HBV | entecavir | J05AF10 | Entecavir Pharmascience International Ltd | 0.5 mg | 30 | 2020-07 | 2026-06 | 4,916 | 301,225 | 61 | Present throughout |
| HBV | entecavir | J05AF10 | Entecavir Alvogen | 0.5 mg | 30 | 2020-07 | 2023-02 | 3,941 | 228,199 | 58 | Present at baseline, withdrawn |
| HBV | entecavir | J05AF10 | Baraclude | 0.5 mg | 30 | 2020-07 | 2024-04 | 427 | 24,933 | 58 | Present at baseline, withdrawn |
| HBV | entecavir | J05AF10 | Entecavir Accord | 0.5 mg | 30 | 2020-07 | 2023-08 | 38 | 2,201 | 58 | Present at baseline, withdrawn |
| HBV | entecavir | J05AF10 | Entecavir Zentiva | 0.5 mg | 30 | 2023-03 | 2026-06 | 7,814 | 493,623 | 63 | Entrant, still supplied |

| Virus | Active substance | ATC | Brand | Strength | Pack size | First month | Last month | Packages | Reimbursement, EUR | Cost per package, EUR | Status |
|---|---|---|---|---|---|---|---|---|---|---|---|
| HBV | lamivudine | J05AF05 | Zeffix | 100 mg | 28 | 2020-07 | 2026-06 | 22,354 | 530,847 | 24 | Present throughout |
| HBV | peginterferon alfa-2a | L03AB11 | Pegasys | 180 mcg-0.5 ml | 1 | 2020-07 | 2026-06 | 1,450 | 229,739 | 158 | Present throughout |
| HBV | telbivudine | J05AF11 | Sebivo | 600 mg | 28 | 2020-07 | 2021-01 | 390 | 155,828 | 400 | Present at baseline, withdrawn |
| HBV | tenofovir disoproxil | J05AF07 | Tenofovir Disoproxil Mylan | 245 mg | 30 | 2020-07 | 2025-04 | 97,886 | 6,979,299 | 71 | Present at baseline, withdrawn |
| HBV | tenofovir disoproxil | J05AF07 | Tenofovir Disoproxil Zentiva | 245 mg | 30 | 2020-07 | 2026-06 | 18,554 | 1,302,386 | 70 | Present throughout |
| HBV | tenofovir disoproxil | J05AF07 | Tenofovir Polpharma | 245 mg | 30 | 2020-07 | 2026-06 | 5,669 | 380,177 | 67 | Present throughout |
| HBV | tenofovir disoproxil | J05AF07 | Virofob | 245 mg | 30 | 2020-07 | 2022-05 | 866 | 71,401 | 82 | Present at baseline, withdrawn |
| HBV | tenofovir disoproxil | J05AF07 | Viread | 245 mg | 30 | 2020-07 | 2021-10 | 405 | 33,488 | 83 | Present at baseline, withdrawn |
| HBV | tenofovir disoproxil | J05AF07 | Tenofovir Disoproxil Accordpharma | 245 mg | - | 2023-11 | 2024-01 | 8 | 499 | 62 | Entrant, since withdrawn |
| HBV | tenofovir disoproxil | J05AF07 | Tenofovir Disoproxil Viatris | 245 mg | 30 | 2025-05 | 2026-06 | 23,164 | 1,556,970 | 67 | Entrant, still supplied |
| HBV | tenofovir disoproxil | J05AF07 | Tenofovir Zentiva | 245 mg | 30 | 2025-11 | 2026-06 | 2,020 | 143,772 | 71 | Entrant, still supplied |
| HCV | elbasvir/grazoprevir | J05AP54 | Zepatier | 50mg/ 100mg | 28 | 2020-07 | 2024-04 | 443 | 3,313,190 | 7,479 | Present at baseline, withdrawn |
| HCV | glecaprevir/pibrentasvir | J05AP57 | Maviret | 100mg/40 mg | 84 | 2020-07 | 2026-06 | 4,718 | 60,482,229 | 12,819 | Present throughout |
| HCV | ledipasvir/sofosbuvir | J05AP51 | Harvoni | 90mg/400 mg | 28 | 2020-07 | 2022-03 | 27 | 387,407 | 14,348 | Present at baseline, withdrawn |

| Virus | Active substance | ATC | Brand | Strength | Pack size | First month | Last month | Packages | Reimbursement, EUR | Cost per package, EUR | Status |
|---|---|---|---|---|---|---|---|---|---|---|---|
| HCV | ribaverin | J05AB04 | Copegus | 200 mg | 168 | 2020-07 | 2021-06 | 63 | 19,499 | 310 | Present at baseline, withdrawn |
| HCV | ribaverin | J05AB04 | Rebetol | 200 mg | 168 | 2020-07 | 2020-07 | 6 | 1,857 | 310 | Present at baseline, withdrawn |
| HCV | sofosbuvir/velpatasvir | J05AP55 | Epclusa | 400 mg/100 mg | 28 | 2020-07 | 2026-06 | 4,743 | 43,293,693 | 9,128 | Present throughout |
| HCV | sofosbuvir/velpatasvir/voxilaprevir | J05AP56 | Vosevi | 400 mg/100mg/100mg | 28 | 2020-07 | 2026-06 | 137 | 1,839,513 | 13,427 | Present throughout |

*Table S3. Fisher decomposition repeated at molecule (ATC) level rather than product level, with the Laspeyres, Paasche and Törnqvist price indices shown alongside the Fisher index. The Törnqvist index reproduces the Fisher index to three decimal places throughout, which indicates that the result does not depend on the choice of index formula.*

| Basis | Virus | Comparison | Expenditure ratio | Fisher price index | Tornqvist price index | Laspeyres price index | Paasche price index | Fisher quantity index | Entry and exit factor |
|---|---|---|---|---|---|---|---|---|---|
| Fixed base | Hepatitis B | 2021 to 2022 | 0.993 | 0.971 | 0.971 | 0.971 | 0.971 | 1.026 | 0.997 |
| | | 2021 to 2023 | 0.860 | 0.784 | 0.784 | 0.785 | 0.783 | 1.101 | 0.997 |
| | | 2021 to 2024 | 0.953 | 0.789 | 0.789 | 0.791 | 0.787 | 1.212 | 0.997 |
| | | 2021 to 2025 | 1.083 | 0.799 | 0.799 | 0.800 | 0.798 | 1.360 | 0.997 |
| | Hepatitis C | 2021 to 2022 | 0.923 | 0.970 | 0.970 | 0.971 | 0.970 | 0.952 | 0.999 |
| | | 2021 to 2023 | 1.180 | 0.943 | 0.943 | 0.943 | 0.944 | 1.267 | 0.987 |
| | | 2021 to 2024 | 1.169 | 0.901 | 0.901 | 0.905 | 0.898 | 1.313 | 0.987 |
| | | 2021 to 2025 | 1.157 | 0.885 | 0.885 | 0.884 | 0.886 | 1.447 | 0.903 |
| Chai | Hep | 2021 to 2022 | 0.993 | 0.971 | 0.971 | 0.971 | 0.971 | 1.026 | 0.997 |
| | | 2022 to 2023 | 0.866 | 0.806 | 0.806 | 0.806 | 0.806 | 1.074 | 1.000 |

| Basis | Virus | Comparison | Expenditure ratio | Fisher price index | Tornqvist price index | Laspeyres price index | Paasche price index | Fisher quantity index | Entry and exit factor |
|---|---|---|---|---|---|---|---|---|---|
| | | 2023 to 2024 | 1.108 | 1.006 | 1.006 | 1.007 | 1.006 | 1.101 | 1.000 |
| | | 2024 to 2025 | 1.136 | 1.012 | 1.012 | 1.011 | 1.012 | 1.123 | 1.000 |
| | Hepatitis C | 2021 to 2022 | 0.923 | 0.970 | 0.970 | 0.971 | 0.970 | 0.952 | 0.999 |
| | | 2022 to 2023 | 1.278 | 0.972 | 0.972 | 0.971 | 0.973 | 1.318 | 0.998 |
| | | 2023 to 2024 | 0.991 | 0.956 | 0.956 | 0.956 | 0.956 | 1.036 | 1.000 |
| | | 2024 to 2025 | 0.990 | 0.980 | 0.980 | 0.982 | 0.979 | 1.011 | 0.999 |

*Table S4. Herfindahl-Hirschman index of reimbursement concentration, computed on cost shares at product and molecule level. Values above 2,500 are conventionally read as a highly concentrated market.*

| Level | Virus | Year | Products or molecules | Herfindahl-Hirschman index |
|---|---|---|---|---|
| Molecule (ATC) | Hepatitis B | 2020 | 5 | 5,414 |
| | | 2021 | 5 | 7,533 |
| | | 2022 | 4 | 7,753 |
| | | 2023 | 4 | 7,308 |
| | | 2024 | 4 | 7,284 |
| | | 2025 | 4 | 7,183 |
| | | 2026 | 4 | 7,356 |
| | Hepatitis C | 2020 | 6 | 4,169 |
| | | 2021 | 6 | 4,105 |
| | | 2022 | 5 | 4,418 |
| | | 2023 | 4 | 4,756 |
| | | 2024 | 4 | 5,197 |

| Level | Virus | Year | Products or molecules | Herfindahl-Hirschman index |
|---|---|---|---|---|
| | | 2025 | 3 | 4,899 |
| | | 2026 | 3 | 5,113 |
| Product (NHIF code) | Hepatitis B | 2020 | 12 | 4,135 |
| | | 2021 | 12 | 5,572 |
| | | 2022 | 9 | 5,492 |
| | | 2023 | 9 | 5,370 |
| | | 2024 | 9 | 5,929 |
| | | 2025 | 8 | 4,425 |
| | | 2026 | 8 | 3,264 |
| | Hepatitis C | 2020 | 7 | 4,169 |
| | | 2021 | 6 | 4,105 |
| | | 2022 | 5 | 4,418 |
| | | 2023 | 4 | 4,756 |
| | | 2024 | 4 | 5,197 |
| | | 2025 | 3 | 4,899 |
| | | 2026 | 3 | 5,113 |

*Table S5. HCV standard-course equivalents and direct-acting antiviral reimbursement per standard course, by regimen. † Partial years: July–December 2020 and January–June 2026. Duration ranges follow the supplementary methods and do not represent statistical uncertainty or unique patients.*

| Year | Regimen | Standard duration, weeks | Packages per standard course | Permitted package range | Standard-course equivalents (range) | Cost per package, EUR | Cost per standard course, EUR (range) |
|---|---|---|---|---|---|---|---|
| 2020 † | elbasvir/grazoprevir | 12 | 3 | 3 to 4 | 32.0 (24.0 to 32.0) | 7,574 | 22,723 (22,723 to 30,297) |
| 2021 | elbasvir/grazoprevir | 12 | 3 | 3 to 4 | 63.0 (47.2 to 63.0) | 7,551 | 22,654 (22,654 to 30,206) |
| 2022 | elbasvir/grazoprevir | 12 | 3 | 3 to 4 | 44.3 (33.2 to 44.3) | 7,390 | 22,170 (22,170 to 29,559) |
| 2023 | elbasvir/grazoprevir | 12 | 3 | 3 to 4 | 7.3 (5.5 to 7.3) | 7,040 | 21,119 (21,119 to 28,158) |
| 2024 | elbasvir/grazoprevir | 12 | 3 | 3 to 4 | 1.0 (0.8 to 1.0) | 7,040 | 21,119 (21,119 to 28,158) |
| 2020 † | glecaprevir/pibrentasvir | 8 | 2 | 2 to 4 | 174.0 (87.0 to 174.0) | 14,406 | 28,812 (28,812 to 57,624) |
| 2021 | glecaprevir/pibrentasvir | 8 | 2 | 2 to 4 | 310.0 (155.0 to 310.0) | 13,963 | 27,926 (27,926 to 55,852) |
| 2022 | glecaprevir/pibrentasvir | 8 | 2 | 2 to 4 | 320.0 (160.0 to 320.0) | 13,305 | 26,611 (26,611 to 53,221) |
| 2023 | glecaprevir/pibrentasvir | 8 | 2 | 2 to 4 | 422.5 (211.2 to 422.5) | 12,764 | 25,528 (25,528 to 51,057) |

| Year | Regimen | Standard duration, weeks | Packages per standard course | Permitted package range | Standard-course equivalents (range) | Cost per package, EUR | Cost per standard course, EUR (range) |
|---|---|---|---|---|---|---|---|
| 2024 | glecaprevir/pibrentasvir | 8 | 2 | 2 to 4 | 500.5 (250.2 to 500.5) | 12,199 | 24,399 (24,399 to 48,797) |
| 2025 | glecaprevir/pibrentasvir | 8 | 2 | 2 to 4 | 412.0 (206.0 to 412.0) | 12,124 | 24,248 (24,248 to 48,496) |
| 2026 † | glecaprevir/pibrentasvir | 8 | 2 | 2 to 4 | 220.0 (110.0 to 220.0) | 12,066 | 24,132 (24,132 to 48,264) |
| 2020 † | ledipasvir/sofosbuvir | 12 | 3 | 2 to 6 | 3.7 (1.8 to 5.5) | 14,406 | 43,218 (28,812 to 86,437) |
| 2021 | ledipasvir/sofosbuvir | 12 | 3 | 2 to 6 | 4.7 (2.3 to 7.0) | 14,321 | 42,962 (28,641 to 85,924) |
| 2022 | ledipasvir/sofosbuvir | 12 | 3 | 2 to 6 | 0.7 (0.3 to 1.0) | 14,226 | 42,677 (28,451 to 85,354) |
| 2020 † | sofosbuvir/velpatasvir | 12 | 3 | 3 to 3 | 90.7 (90.7 to 90.7) | 9,538 | 28,613 (28,613 to 28,613) |
| 2021 | sofosbuvir/velpatasvir | 12 | 3 | 3 to 3 | 220.3 (220.3 to 220.3) | 9,538 | 28,613 (28,613 to 28,613) |
| 2022 | sofosbuvir/velpatasvir | 12 | 3 | 3 to 3 | 204.0 (204.0 to 204.0) | 9,472 | 28,415 (28,415 to 28,415) |
| 2023 | sofosbuvir/velpatasvir | 12 | 3 | 3 to 3 | 300.7 (300.7 to 300.7) | 9,395 | 28,185 (28,185 to 28,185) |

| Year | Regimen | Standard duration, weeks | Packages per standard course | Permitted package range | Standard-course equivalents (range) | Cost per package, EUR | Cost per standard course, EUR (range) |
|---|---|---|---|---|---|---|---|
| 2024 | sofosbuvir/velpatasvir | 12 | 3 | 3 to 3 | 269.7 (269.7 to 269.7) | 8,983 | 26,949 (26,949 to 26,949) |
| 2025 | sofosbuvir/velpatasvir | 12 | 3 | 3 to 3 | 359.0 (359.0 to 359.0) | 8,646 | 25,938 (25,938 to 25,938) |
| 2026 † | sofosbuvir/velpatasvir | 12 | 3 | 3 to 3 | 136.7 (136.7 to 136.7) | 8,646 | 25,938 (25,938 to 25,938) |
| 2020 † | sofosbuvir/velpatasvir/voxilaprevir | 12 | 3 | 3 to 3 | 7.7 (7.7 to 7.7) | 14,406 | 43,218 (43,218 to 43,218) |
| 2021 | sofosbuvir/velpatasvir/voxilaprevir | 12 | 3 | 3 to 3 | 6.3 (6.3 to 6.3) | 14,205 | 42,616 (42,616 to 42,616) |
| 2022 | sofosbuvir/velpatasvir/voxilaprevir | 12 | 3 | 3 to 3 | 6.0 (6.0 to 6.0) | 13,862 | 41,587 (41,587 to 41,587) |
| 2023 | sofosbuvir/velpatasvir/voxilaprevir | 12 | 3 | 3 to 3 | 12.3 (12.3 to 12.3) | 13,184 | 39,552 (39,552 to 39,552) |

| Year | Regimen | Standard duration, weeks | Packages per standard course | Permitted package range | Standard-course equivalents (range) | Cost per package, EUR | Cost per standard course, EUR (range) |
|---|---|---|---|---|---|---|---|
| 2024 | sofosbuvir/velpatasvir/voxilaprevir | 12 | 3 | 3 to 3 | 5.7 (5.7 to 5.7) | 12,636 | 37,907 (37,907 to 37,907) |
| 2025 | sofosbuvir/velpatasvir/voxilaprevir | 12 | 3 | 3 to 3 | 5.7 (5.7 to 5.7) | 12,485 | 37,455 (37,455 to 37,455) |
| 2026 † | sofosbuvir/velpatasvir/voxilaprevir | 12 | 3 | 3 to 3 | 2.0 (2.0 to 2.0) | 12,315 | 36,944 (36,944 to 36,944) |

*Table S6. Hospital admissions in which hepatitis B or C was recorded as the secondary diagnosis, by primary discharge diagnosis, for diagnoses with at least 20 admissions. These admissions are reported separately throughout and are never added to admissions for hepatitis itself.*

| Virus recorded as secondary diagnosis | Primary discharge diagnosis (ICD-10) | Admissions |
|---|---|---|
| HBV | K74.6 | 3,029 |
| HCV | K74.6 | 765 |
| HBV | K76.0 | 364 |
| HBV | K80.8 | 315 |
| HBV | K82.8 | 303 |
| HBV | Z94.4 | 248 |
| HCV | D56.1 | 198 |
| HCV | K76.0 | 173 |
| HBV | D13.4 | 147 |

| Virus recorded as secondary diagnosis | Primary discharge diagnosis (ICD-10) | Admissions |
|---|---|---|
| HCV | D66 | 132 |
| HBV | C22.0 | 104 |
| HBV | M45.0 | 100 |
| HCV | K82.8 | 99 |
| HBV | C83.3 | 96 |
| HBV | M05.80 | 86 |
| HBV | L40.0 | 77 |
| HCV | K80.8 | 64 |
| HBV | C90.0 | 56 |
| HBV | K80.1 | 50 |
| HBV | C91.1 | 45 |
| HCV | D67 | 43 |
| HBV | K51.8 | 38 |
| Unspecified | C90.0 | 38 |
| HBV | D18.0 | 37 |
| Unspecified | J96.0 | 35 |
| HBV | D56.1 | 32 |
| HBV | M51.1 | 32 |
| HCV | M45.0 | 32 |
| HCV | D13.4 | 31 |
| HBV | C50.4 | 29 |
| HBV | C82.0 | 29 |
| HBV | K50.0 | 28 |
| HCV | L40.0 | 28 |
| HBV | E11.4 | 27 |

| Virus recorded as secondary diagnosis | Primary discharge diagnosis (ICD-10) | Admissions |
|---|---|---|
| HCV | C22.0 | 25 |
| HBV | C78.7 | 24 |
| HBV | L40.5 | 22 |
| HBV | C82.7 | 21 |
| HBV | R40.0 | 21 |
| Unspecified | K74.6 | 21 |
| HBV | J15.8 | 20 |
| HBV | K72.0 | 20 |
| Unspecified | M51.1 | 20 |

*Table S7. Clinical pathways, ambulatory procedures and clinical procedures under which hepatitis admissions were billed, for codes accounting for at least 0.3% of admissions. "A" codes are ambulatory procedures, "P" codes clinical pathways and "K" codes clinical procedures; the A99 suffix is an NHIF billing variant.*

| Virus | Pathway or procedure code | Admissions | Share of admissions, % | Contracted facilities |
|---|---|---|---|---|
| Hepatitis B | A38 | 14,449 | 44.8 | 19 |
| | P076.1 | 11,667 | 36.2 | 72 |
| | P076.1A99 | 4,330 | 13.4 | 36 |
| | P073.1 | 1,035 | 3.2 | 31 |
| | P101 | 329 | 1.0 | 36 |
| | P073.1A99 | 284 | 0.9 | 11 |
| Hepatitis C | P076.1 | 2,411 | 44.4 | 59 |
| | A38 | 1,777 | 32.8 | 19 |
| | P076.1A99 | 935 | 17.2 | 26 |
| | P073.1 | 154 | 2.8 | 15 |

| Virus | Pathway or procedure code | Admissions | Share of admissions, % | Contracted facilities |
|---|---|---|---|---|
| | P101 | 99 | 1.8 | 29 |
| | P073.1A99 | 27 | 0.5 | 5 |

*Table S8. Nominal and constant-2025-price antiviral reimbursement, deflated by the Bulgarian all-items harmonised index of consumer prices. The all-items index was used in preference to the health sub-index, which contains an 11% level shift between March and April 2025 that would propagate into any series spanning it.*

| Year | Virus | Nominal, EUR | HICP index (2015 = 100) | Constant 2025 prices, EUR |
|---|---|---|---|---|
| 2020 † | Hepatitis B | 1,176,173 | 106.3 | 1,577,158 |
| 2021 | | 2,037,503 | 109.3 | 2,656,397 |
| 2022 | | 2,022,898 | 123.5 | 2,333,735 |
| 2023 | | 1,752,227 | 134.2 | 1,861,293 |
| 2024 | | 1,941,433 | 137.6 | 2,010,130 |
| 2025 | | 2,205,974 | 142.5 | 2,205,974 |
| 2026 † | | 1,298,379 | 147.7 | 1,252,837 |
| 2020 † | Hepatitis C | 8,833,125 | 106.3 | 11,844,550 |
| 2021 | | 16,871,845 | 109.3 | 21,996,687 |
| 2022 | | 15,572,863 | 123.5 | 17,965,779 |
| 2023 | | 19,902,588 | 134.2 | 21,141,400 |
| 2024 | | 19,714,761 | 137.6 | 20,412,363 |

| Year | Virus | Nominal, EUR | HICP index (2015 = 100) | Constant 2025 prices, EUR |
|---|---|---|---|---|
| 2025 | | 19,514,347 | 142.5 | 19,514,347 |
| 2026 † | | 8,927,858 | 147.7 | 8,614,706 |

*Table S9. Adjunctive products under HBV/HCV diagnoses in the full pharmacy window, July 2020–June 2026. Their observed total is EUR 113,942, approximately 0.09% of all reimbursement in the HBV/HCV pharmacy case definition. These amounts enter the broader pharmaceutical budget-share numerator, while antiviral-only totals exclude them.*

| Virus diagnosis | Active substance | Packages | Reimbursement, EUR |
|---|---|---|---|
| HBV | ademetionine | 9,213 | 35,554 |
| HBV | silymarin | 22,132 | 32,780 |
| HCV | silymarin | 14,748 | 20,734 |
| HCV | ademetionine | 4,574 | 17,225 |
| HBV | l-ornitine-l-aspartate | 404 | 4,334 |
| HCV | l-ornitine-l-aspartate | 316 | 3,315 |

*Table S10. Antiviral reimbursement by regional health insurance office, 2021 to 2025, in absolute terms and per 100,000 person-years. Activity is attributed to the office that processed the claim rather than to the patient's residence, which is why Sofia city and Sofia district should be read together.*

| Region | Virus | Reimbursement 2021-2025, EUR | Per 100,000 person-years, EUR |
|---|---|---|---|
| Blagoevgrad | Hep | 776,261 | 54,003 |
| Burgas | | 688,950 | 35,836 |

| Region | Virus | Reimbursement 2021-2025, EUR | Per 100,000 person-years, EUR |
|---|---|---|---|
| Dobrich | | 273,396 | 37,354 |
| Gabrovo | | 95,830 | 20,185 |
| Haskovo | | 343,978 | 33,139 |
| Kardzhali | | 278,750 | 38,113 |
| Kyustendil | | 100,045 | 18,611 |
| Lovech | | 139,478 | 24,863 |
| Montana | | 110,566 | 19,312 |
| Pazardzhik | | 398,291 | 35,443 |
| Pernik | | 75,339 | 13,579 |
| Pleven | | 301,584 | 27,669 |
| Plovdiv | | 1,130,387 | 35,650 |
| Razgrad | | 161,496 | 32,211 |
| Ruse | | 265,813 | 28,275 |
| Shumen | | 177,473 | 23,738 |
| Silistra | | 164,141 | 34,695 |
| Sliven | | 409,709 | 48,218 |
| Smolyan | | 110,919 | 24,115 |
| Sofia Grad | | 1,775,286 | 27,560 |
| Sofia Oblast | | 185,837 | 16,419 |
| Stara Zagora | | 523,323 | 36,038 |
| Targovishte | | 92,779 | 19,442 |
| Varna | | 639,069 | 29,392 |
| Veliko Tarnovo | | 300,163 | 29,664 |

| Region | Virus | Reimbursement 2021-2025, EUR | Per 100,000 person-years, EUR |
|---|---|---|---|
| Vidin | | 94,712 | 26,399 |
| Vratsa | | 186,575 | 25,280 |
| Yambol | | 159,888 | 30,034 |
| Blagoevgrad | Hepatitis C | 3,540,902 | 246,335 |
| Burgas | | 4,356,150 | 226,590 |
| Dobrich | | 1,597,356 | 218,245 |
| Gabrovo | | 803,692 | 169,282 |
| Haskovo | | 1,370,119 | 131,999 |
| Kardzhali | | 939,352 | 128,437 |
| Kyustendil | | 1,174,402 | 218,473 |
| Lovech | | 1,237,923 | 220,665 |
| Montana | | 1,656,876 | 289,402 |
| Pazardzhik | | 3,213,297 | 285,940 |
| Pernik | | 1,550,143 | 279,397 |
| Pleven | | 3,090,425 | 283,537 |
| Plovdiv | | 10,113,604 | 318,962 |
| Razgrad | | 1,308,996 | 261,082 |
| Ruse | | 3,400,686 | 361,732 |
| Shumen | | 1,402,396 | 187,581 |
| Silistra | | 1,542,764 | 326,102 |
| Sliven | | 2,557,675 | 301,011 |
| Smolyan | | 825,297 | 179,427 |
| Sofia Grad | | 27,122,345 | 421,051 |

| Region | Virus | Reimbursement 2021-2025, EUR | Per 100,000 person-years, EUR |
|---|---|---|---|
| Sofia Oblast | | 1,051,334 | 92,887 |
| Stara Zagora | | 3,102,814 | 213,673 |
| Targovishte | | 513,932 | 107,698 |
| Varna | | 6,868,754 | 315,907 |
| Veliko Tarnovo | | 2,411,381 | 238,308 |
| Vidin | | 979,908 | 273,129 |
| Vratsa | | 2,365,835 | 320,562 |
| Yambol | | 1,478,047 | 277,644 |

*Table S11. Interrupted time series at January 2026, when Bulgaria adopted the euro and, the following month, the NHIF revised its treatment requirements. Quasi-Poisson segmented regression with harmonic seasonal terms and Newey–West standard errors at lag 3, on 66 pre-intervention and 6 post-intervention months. With only six post-intervention months and two coincident events, these estimates are provisional.*

| **Virus** | **Parameter** | **Ratio (95% CI)** | **p** |
|---|---|---|---|
| Hepatitis B | Pre-intervention monthly trend | 1.000 (0.997 to 1.002) | 0.694 |
| | Level change at January 2026 | 1.262 (1.086 to 1.467) | 0.003 |
| | Change in monthly trend | 1.014 (0.989 to 1.040) | 0.256 |
| Hepatitis C | Pre-intervention monthly trend | 1.003 (1.001 to 1.005) | 0.003 |
| | Level change at January 2026 | 0.777 (0.678 to 0.891) | <0.001 |
| | Change in monthly trend | 1.016 (0.985 to 1.047) | 0.308 |

*Table S12. Hepatitis hospital activity valued at the 2025 National Framework Contract tariff schedule. One dated schedule is applied to every year deliberately: tariffs are revised annually and amended within years, and only the 2025 schedule could be sourced completely, so this series moves with volume alone and is not a reconstruction of what the Fund paid in each year. It is never added to pharmacy expenditure.*

| Year | Virus | Episode type | Episodes | Episodes priced, % | Valued at 2025 tariffs, EUR |
|---|---|---|---|---|---|
| 2024 | Hepatitis B | Inpatient episode | 6,504 | 99.6 | 4,594,601 |
| 2024 | Hepatitis B | Treatment-protocol procedure | 5,044 | 100.0 | 154,750 |
| 2024 | Hepatitis C | Inpatient episode | 1,496 | 99.5 | 1,047,934 |
| 2024 | Hepatitis C | Treatment-protocol procedure | 708 | 100.0 | 21,721 |
| 2025 | Hepatitis B | Inpatient episode | 7,020 | 99.7 | 4,884,691 |
| 2025 | Hepatitis B | Treatment-protocol procedure | 5,646 | 100.0 | 173,219 |
| 2025 | Hepatitis C | Inpatient episode | 1,401 | 99.9 | 958,707 |
| 2025 | Hepatitis C | Treatment-protocol procedure | 684 | 100.0 | 20,985 |
| 2026 | Hepatitis B | Inpatient episode | 4,296 | 99.2 | 2,864,793 |
| 2026 | Hepatitis B | Treatment-protocol procedure | 3,759 | 100.0 | 115,326 |
| 2026 | Hepatitis C | Inpatient episode | 751 | 99.9 | 488,909 |
| 2026 | Hepatitis C | Treatment-protocol procedure | 385 | 100.0 | 11,812 |

*Table S13. The 2025 tariffs applied, by pathway and procedure code. Codes carrying the A99 suffix are cases reported as ambulatory procedure 99, the early completion of a clinical pathway: Article 354(1) of the National Framework Contract allows a pathway with a contracted minimum stay of up to three days to be closed this way once all mandatory activities have been performed after at least twelve hours and a physician has recorded that early discharge carries no medical risk. Such a case is paid at 80% of the pathway price, and is valued here at that rate; the exceptions in the contract — 60% for pathways 240 to 243 and 245, and the full price for pathways 16, 17.1, 17.2, 19.1, 20.1, 20.2, 25, 27 and 33 — cover no hepatitis pathway [40]. Ambulatory procedure 38 is priced at EUR 30.68 from the same schedule. Codes accounting for 0.2% of episodes could not be priced at all.*

| Virus | Code | Pathway or procedure | 2025 tariff, EUR | Episodes | Valued, EUR |
|---|---|---|---|---|---|
| HBV | A38 | Determination of treatment plan and monitoring of therapeutic response in patients receiving high-cost medicinal products under Article 78(2) of the Health Insurance Act | 31 | 14,449 | 443,295 |
| HBV | P076.1 | Diagnosis and treatment of chronic liver disease in persons aged 18 and over | 668 | 11,667 | 7,795,423 |
| HBV | P076.1A99 | Diagnosis and treatment of chronic liver disease in persons aged 18 and over, early completion (APr 99) | 535 | 4,330 | 2,314,515 |
| HCV | P076.1 | Diagnosis and treatment of chronic liver disease in persons aged 18 and over | 668 | 2,411 | 1,610,934 |
| HCV | A38 | Determination of treatment plan and monitoring of therapeutic response in patients receiving high-cost medicinal products under Article 78(2) of the Health Insurance Act | 31 | 1,777 | 54,518 |
| HBV | P073.1 | Highly specialised interventional procedures for hepatobiliary, pancreatic and peritoneal disease, 18 and over | 1,338 | 1,035 | 1,384,892 |
| HCV | P076.1A99 | Diagnosis and treatment of chronic liver disease in persons aged 18 and over, early completion (APr 99) | 535 | 935 | 499,786 |
| HBV | P101 | Diagnosis and treatment of acute viral hepatitis B, C and D | 1,416 | 329 | 465,917 |
| HBV | P073.1A99 | Highly specialised interventional procedures for hepatobiliary, pancreatic and peritoneal disease, 18 and over, early completion (APr 99) | 1,070 | 284 | 304,008 |
| HCV | P073.1 | Highly specialised interventional procedures for hepatobiliary, pancreatic and peritoneal disease, 18 and over | 1,338 | 154 | 206,061 |
| HCV | P101 | Diagnosis and treatment of acute viral hepatitis B, C and D | 1,416 | 99 | 140,200 |
| HBV | P076.2 | Diagnosis and treatment of chronic liver disease in persons under 18 | 907 | 66 | 59,867 |
| HCV | P073.1A99 | Highly specialised interventional procedures for hepatobiliary, pancreatic and peritoneal disease, 18 and over, early completion (APr 99) | 1,070 | 27 | 28,902 |
| HBV | P076.2A99 | Diagnosis and treatment of chronic liver disease in persons under 18, early completion (APr 99) | 726 | 26 | 18,867 |
| HCV | P076.2 | Diagnosis and treatment of chronic liver disease in persons under 18 | 907 | 6 | 5,442 |

| Virus | Code | Pathway or procedure | 2025 tariff, EUR | Episodes | Valued, EUR |
|---|---|---|---|---|---|
| HCV | P076.2A99 | Diagnosis and treatment of chronic liver disease in persons under 18, early completion (APr 99) | 726 | 5 | 3,628 |
| HBV | P090.1 | Diagnosis and treatment of inflammatory joint disease in persons aged 18 and over | 596 | 1 | 596 |
| HCV | P090.1 | Diagnosis and treatment of inflammatory joint disease in persons aged 18 and over | 596 | 1 | 596 |

*Table S14. Treatment-protocol procedures in the full January 2024–July 2026 source window. These are repeated administrative encounters within the same reimbursement system, not unique patients or an independent validation of dispensing-based estimates. The 31-month totals annualise to 5,593 HBV and 688 HCV procedures. In complete years 2024–2025 the corresponding observed annual means were 5,345 and 696.*

| Virus | Procedures observed | Months | Designated facilities | Procedures per year |
|---|---|---|---|---|
| Hepatitis B | 14,449 | 31 | 19 | 5,593 |
| Hepatitis C | 1,777 | 31 | 19 | 688 |

*Table S15. National Health Insurance Fund data sources analysed, their coverage and the number of records meeting the hepatitis case definition.*

| Data source | Coverage | Mon ths | Aggregation unit | Records in scope |
|---|---|---|---|---|
| Outpatient pharmacy reimbursement | Jul 2020 to Jun 2026 | 72 | product x region x diagnosis x ten-day sub-period | 29,151 |
| Hospitalisations | Jan 2024 to Jul 2026 | 31 | facility x pathway x diagnosis pair x month | 22,151 |
| Outpatient consultations | Jan 2024 to Jul 2026 | 31 | practice x specialty x diagnosis x month | 45,304 |

*Table S16. Full-window annual antiviral reimbursement and dispensing-based treatment estimates, July 2020–June 2026. † Partial years: July–December 2020 and January–June 2026. The budget-share numerator is all reimbursement in the HBV/HCV pharmacy case definition, comprising antivirals in both identification arms plus adjunctive products; the denominator is all NHIF outpatient pharmaceutical reimbursement in the same observed months. This is not an antiviral-only share. Complete-year primary results are in manuscript Table 1.*

| Year | Hepatitis B, EUR | HBV mean continuous therapy equivalents | Hepatitis C, EUR | Hepatitis C, standard-course equivalents (range) | All hepatitis products share of NHIF outpatient drug budget, % |
|---|---|---|---|---|---|
| 2020 † | 1,176,173 | 2,224 | 8,833,125 | 308 (211 to 310) | 4.34 |
| 2021 | 2,037,503 | 2,226 | 16,871,845 | 604 (431 to 607) | 3.99 |
| 2022 | 2,022,898 | 2,277 | 15,572,863 | 575 (404 to 575) | 3.33 |
| 2023 | 1,752,227 | 2,441 | 19,902,588 | 743 (530 to 743) | 3.48 |
| 2024 | 1,941,433 | 2,675 | 19,714,761 | 777 (526 to 777) | 3.06 |
| 2025 | 2,205,974 | 3,051 | 19,514,347 | 777 (571 to 777) | 2.61 |
| 2026 † | 1,298,379 | 3,182 | 8,927,858 | 359 (249 to 359) | 2.26 |

*Table S17. Log-linear monthly monetary-expenditure sensitivity models, with annual sine/cosine terms and Bartlett Newey–West covariance at lag 3. Primary windows contain 60 months (2021–2025); full windows contain 72 months (July 2020–June 2026). All rows use normal Wald confidence intervals and p-values for a consistent comparison.*

| Virus | Months | Monthly ratio (95% CI) | Annualised change, % | p |
|---|---|---|---|---|
| HBV 2021–2025 | 60 | 1.0010 (0.9992 to 1.0029) | +1.2 | 0.285 |
| HBV full window | 72 | 1.0012 (0.9987 to 1.0037) | +1.5 | 0.332 |
| HCV 2021–2025 | 60 | 1.0046 (1.0027 to 1.0065) | +5.7 | <0.001 |
| HCV full window | 72 | 1.0019 (1.0002 to 1.0037) | +2.4 | 0.031 |

# Supplementary figures

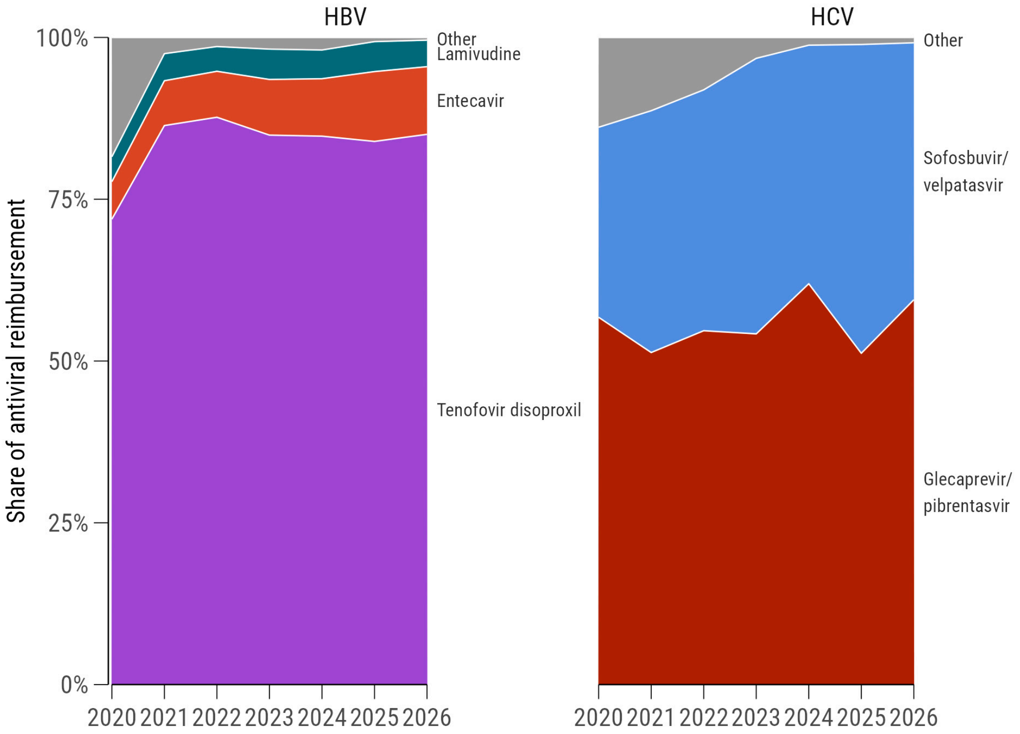


*Figure S1. Annual composition of antiviral reimbursement by leading product. Products outside the displayed categories are combined as Other.*

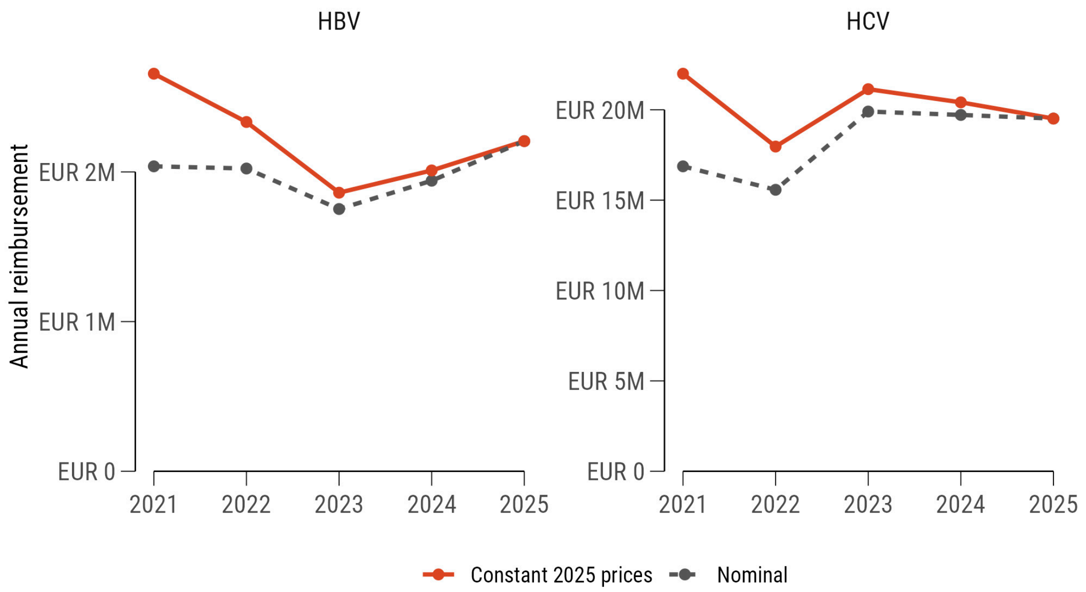


*Figure S2. Nominal and constant-2025-price antiviral reimbursement, 2021 to 2025.*

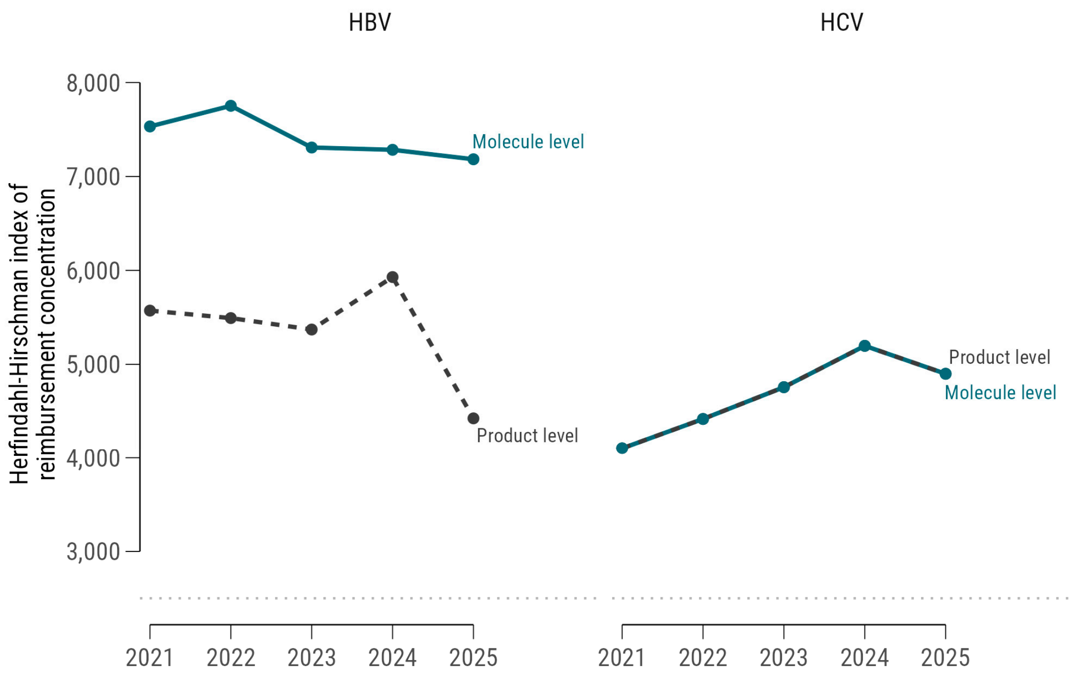


*Figure S3. Herfindahl-Hirschman index of reimbursement concentration by virus and year, at product and molecule level. The shaded band begins at 2,500.*

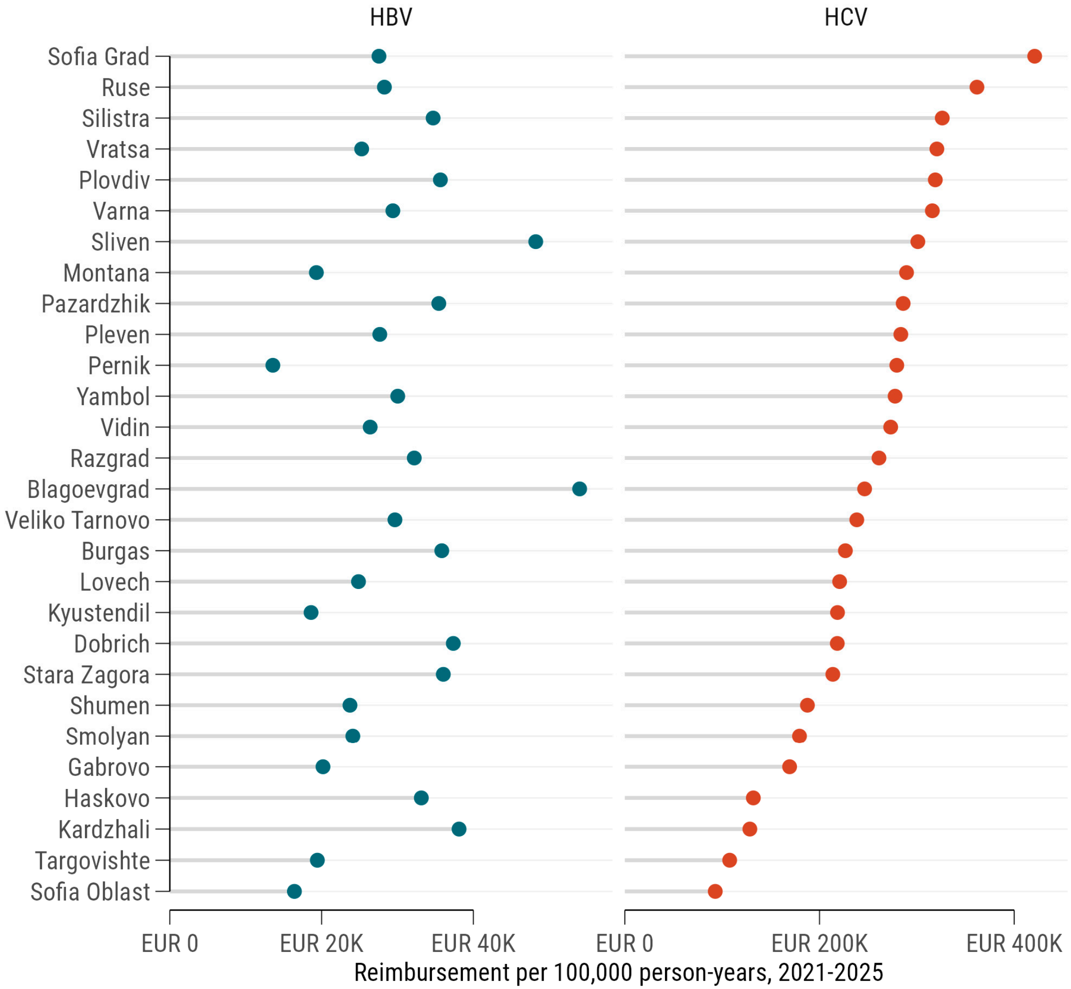


*Figure S4. Antiviral reimbursement per 100,000 person-years by processing regional health insurance office, 2021 to 2025. The office is not necessarily the patient's place of residence.*

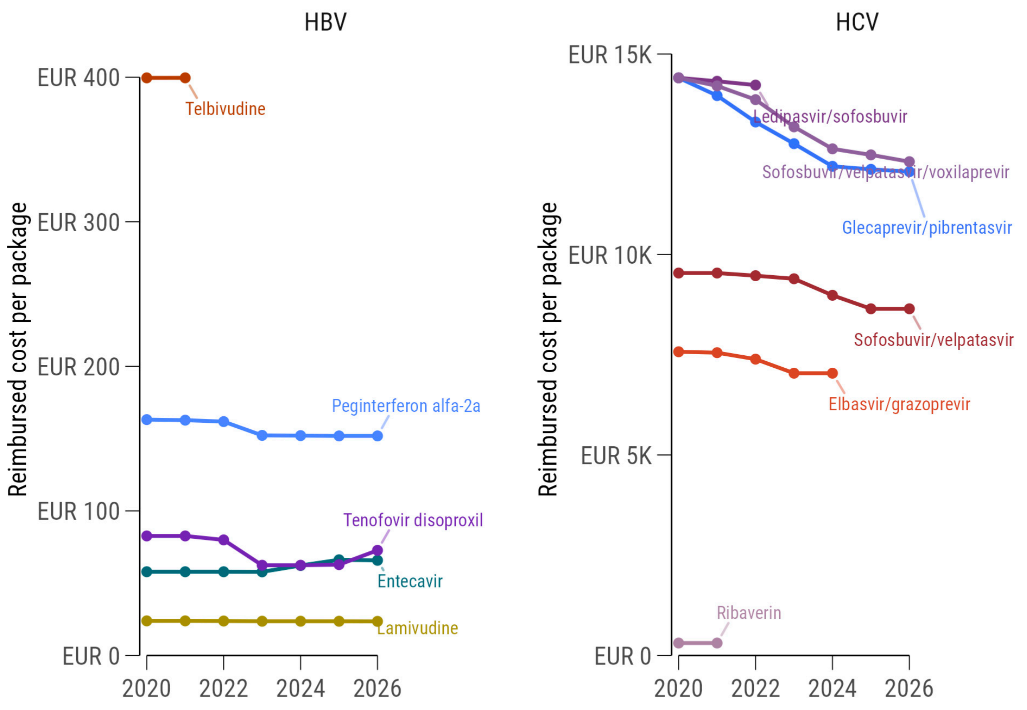


*Figure S5. Annual reimbursed cost per package for the leading antiviral molecules. Values for 2020 and 2026 cover partial years.*

# Tables S18 and S19. Reporting checklists

The study is reported in accordance with the STROBE statement for observational studies [31], extended by RECORD for studies using routinely collected health data [32]. Items are located by subheading rather than by page number, since page numbers change between the submitted and typeset versions.

## STROBE checklist

*Table S18. STROBE checklist.*

| Item | Checklist item | Reported in |
|---|---|---|
| 1 | (a) Indicate the study's design with a commonly used term in the title or the abstract. (b) Provide in the abstract an informative and balanced summary of what was done and what was found | Title; Abstract → Background, Methods, Results, Conclusions |
| 2 | Explain the scientific background and rationale for the investigation being reported | Introduction |
| 3 | State specific objectives, including any prespecified hypotheses | Introduction, final paragraph. No hypotheses were prespecified; the study is descriptive |
| 4 | Present key elements of study design early in the paper | Abstract → Methods; Materials and Methods → Study design and reporting |
| 5 | Describe the setting, locations, and relevant dates, including periods of recruitment, exposure, follow-up, and data collection | Materials and Methods → Data sources; Table S15 |
| 6 | (a) Cross-sectional study — Give the eligibility criteria, and the sources and methods of selection of participants | Materials and Methods → Case definition; Supplementary methods → Case definition in full. (b) N/A — not a matched study |
| 7 | Clearly define all outcomes, exposures, predictors, potential confounders, and effect modifiers. Give diagnostic criteria, if applicable | Materials and Methods → Case definition; Expenditure and price adjustment; Treatment volumes. Confounders and effect modifiers N/A — no causal estimand is targeted |
| 8 | For each variable of interest, give sources of data and details of methods of assessment | Materials and Methods → Data sources; Expenditure and price adjustment; Treatment volumes |
| 9 | Describe any efforts to address potential sources of bias | Supplementary methods → Data quality and corrections; Discussion → Strengths and limitations |
| 10 | Explain how the study size was arrived at | Materials and Methods → Data sources. The study is a total enumeration of national reimbursement records; no sampling and no size calculation |

| Item | Checklist item | Reported in |
| --- | --- | --- |
| 11 | Explain how quantitative variables were handled in the analyses. If applicable, describe which groupings were chosen and why | Materials and Methods → Expenditure and price adjustment; Treatment volumes; Statistical analysis |
| 12 | (a) Describe all statistical methods, including those used to control for confounding. (b) subgroups and interactions. (c) missing data. (d) sampling strategy. (e) sensitivity analyses | (a), (b), (e) Materials and Methods → Price–volume decomposition; Statistical analysis. (c) Not reported as a separate item — the aggregated source contains no missing reimbursement or count fields within the hepatitis subset; the one missing package size is described in Supplementary methods. (d) N/A — total enumeration, no sampling |
| 13 | (a) Report numbers of individuals at each stage. (b) Give reasons for non-participation. (c) Consider use of a flow diagram | Table S15 gives records at each source. (a)–(c) partly N/A — the data are aggregated counts, not individuals, so no individual-level flow exists and no flow diagram is possible |
| 14 | (a) Give characteristics of study participants and information on exposures and potential confounders. (b) missing data per variable. (c) follow-up time | N/A — aggregated data with no participant characteristics. Distribution across the case definition is in Supplementary Table S1 |
| 15 | Report numbers of outcome events or summary measures over time | Results → Records and reimbursed expenditure; Price, quantity, and treatment volume; Hospital and outpatient activity |
| 16 | (a) Give unadjusted and, if applicable, confounder-adjusted estimates and their precision. (b) category boundaries. (c) absolute risk | (a) Results → Price, quantity, and treatment volume; Hospital and outpatient activity; Table 5 and Table S20, with 95% confidence intervals for model estimates. No adjustment for confounding is made and none is claimed. (b) N/A. (c) N/A |
| 17 | Report other analyses done, including sensitivity analyses | Supplementary Tables S3, S4, S8, S10, S11, and S17 |
| 18 | Summarise key results with reference to study objectives | Discussion → Principal findings |
| 19 | Discuss limitations of the study, taking into account sources of potential bias or imprecision. Discuss both direction and magnitude of any potential bias | Discussion → Strengths and limitations |

| Item | Checklist item | Reported in |
|---|---|---|
| 20 | Give a cautious overall interpretation of results considering objectives, limitations, multiplicity of analyses, results from similar studies, and other relevant evidence | Discussion → Principal findings; Comparison with other evidence; Implications; Conclusions |
| 21 | Discuss the generalisability (external validity) of the study results | Discussion → Comparison with other evidence; Strengths and limitations |
| 22 | Give the source of funding and the role of the funders | Funding |

# RECORD checklist

*Table S19. RECORD checklist.*

| Item | Checklist item | Reported in |
|---|---|---|
| 1.1 | The type of data used should be specified in the title or abstract. When possible, the name of the databases used should be included | Title ("Health Insurance Expenditure and Health-System Use"); Abstract → Methods, which names the three National Health Insurance Fund datasets |
| 1.2 | If applicable, the geographical region and timeframe within which the study took place should be reported in the title or abstract | Title ("Bulgaria, 2021–2025"); Abstract → Methods |
| 1.3 | If linkage between databases was conducted for the study, this should be clearly stated in the title or abstract | N/A — no linkage. The three datasets are analysed in parallel and are never joined to one another; the only joins are to aggregate population and price-index denominators |
| 6.1 | The methods of study population selection (such as codes or algorithms used to identify participants) should be listed in detail. If this is not possible, an explanation should be provided | Materials and Methods → Case definition; Supplementary methods → Case definition in full gives every ICD-10 code and its group |
| 6.2 | Any validation studies of the codes or algorithms used to select the population should be referenced. If validation was conducted for this study and not published elsewhere, detailed methods and results should be provided | Not reported — no validation study of NHIF hepatitis diagnosis coding against clinical records exists to our knowledge, and none was conducted here. Stated as a limitation in Discussion → Strengths and limitations |

| Item | Checklist item | Reported in |
|---|---|---|
| 6.3 | If the study involved linkage of databases, consider use of a flow diagram or other graphical display to demonstrate the data linkage process | N/A — no linkage, as at item 1.3 |
| 7.1 | A complete list of codes and algorithms used to classify exposures, outcomes, confounders, and effect modifiers should be provided. If these cannot be reported, an explanation should be provided | Materials and Methods → Case definition; Expenditure and price adjustment (ATC classification rules); Supplementary methods → Case definition in full; Treatment course definitions |
| 12.1 | Authors should describe the extent to which the investigators had access to the database population used to create the study population | Materials and Methods → Data sources; Data Availability Statement. All three datasets are openly published in full; the investigators had complete access to every released record |
| 12.2 | Authors should provide information on the data cleaning methods used in the study | Supplementary methods → Data quality and corrections |
| 12.3 | State whether the study included person level, institutional level, or other data linkage across two or more databases | Materials and Methods → Data sources. No linkage; the data are aggregated counts, not person-level records |
| 13.1 | Describe in detail the selection of the individuals included in the study including filtering based on data quality, data availability, and linkage | Materials and Methods → Case definition; Supplementary methods → Data quality and corrections; Table S15. Selection is at record rather than individual level, since the source is aggregated |
| 19.1 | Discuss the implications of using data that were not created or collected to answer the specific research question(s). Include discussion of misclassification bias, unmeasured confounding, missing data, and changing eligibility over time | Discussion → Strengths and limitations, which covers reimbursement-driven coding, the absence of individual-level follow-up, costs falling outside the NHIF, and the coincidence of the euro changeover with the February 2026 revision of treatment eligibility |
| 22.1 | Authors should provide information on how to access any supplemental information such as the study protocol, raw data, or programming code | Data Availability Statement, which gives the DOI of each source dataset and states how the analysis code may be obtained |

# Additional sensitivity analyses

Table S20A. Complete-year and full-window seasonal quasi-Poisson models. Reimbursement windows contain 60 and 72 months, respectively; service windows contain 24 and 31. Bartlett covariance lag 3 and normal Wald inference are used throughout.

| **Series** | **Window** | **Months** | **Monthly ratio and 95% CI** | **Annualised change %** | **p** |
|---|---|---|---|---|---|
| Antiviral reimbursement HBV | Complete years | 60 | 1.0011 (0.9993–1.0030) | +1.4 | 0.219 |
| Antiviral reimbursement HBV | Full source window | 72 | 1.0014 (0.9988–1.0040) | +1.7 | 0.283 |
| Antiviral reimbursement HCV | Complete years | 60 | 1.0044 (1.0025–1.0063) | +5.4 | <0.001 |
| Antiviral reimbursement HCV | Full source window | 72 | 1.0018 (1.0001–1.0035) | +2.2 | 0.042 |
| Inpatient episodes HBV | Complete years | 24 | 1.0074 (1.0030–1.0118) | +9.2 | <0.001 |
| Inpatient episodes HBV | Full source window | 31 | 1.0060 (1.0034–1.0087) | +7.5 | <0.001 |
| Inpatient episodes HCV | Complete years | 24 | 0.9936 (0.9898–0.9974) | -7.4 | <0.001 |
| Inpatient episodes HCV | Full source window | 31 | 0.9935 (0.9913–0.9958) | -7.5 | <0.001 |
| Protocol procedures HBV | Complete years | 24 | 1.0100 (1.0053–1.0147) | +12.7 | <0.001 |
| Protocol procedures HBV | Full source window | 31 | 1.0113 (1.0082–1.0143) | +14.4 | <0.001 |
| Protocol procedures HCV | Complete years | 24 | 0.9958 (0.9889–1.0026) | -5.0 | 0.225 |
| Protocol procedures HCV | Full source window | 31 | 0.9979 (0.9928–1.0030) | -2.5 | 0.426 |
| Consultations HBV | Complete years | 24 | 1.0030 (1.0012–1.0048) | +3.7 | <0.001 |
| Consultations HBV | Full source window | 31 | 1.0029 (1.0018–1.0040) | +3.5 | <0.001 |
| Consultations HCV | Complete years | 24 | 0.9941 (0.9904–0.9978) | -6.9 | 0.002 |
| Consultations HCV | Full source window | 31 | 0.9948 (0.9926–0.9971) | -6.1 | <0.001 |

Table S20B. Complete-year sensitivity to seasonal specification and covariance lag. Removing seasonal terms changes the point estimate; changing only the covariance lag changes uncertainty without changing the estimated trend. Lag 0 is the heteroskedasticity-robust limit of the Bartlett estimator.

| Series | Specification | HAC lag | Monthly ratio and 95% CI | Annualised change % | p |
|---|---|---|---|---|---|
| Antiviral reimbursement HBV | Annual sine/cosine | 0 | 1.0011 (0.9999–1.0024) | +1.4 | 0.068 |
| Antiviral reimbursement HBV | Annual sine/cosine | 1 | 1.0011 (0.9997–1.0026) | +1.4 | 0.124 |
| Antiviral reimbursement HBV | Annual sine/cosine | 6 | 1.0011 (0.9990–1.0033) | +1.4 | 0.301 |
| Antiviral reimbursement HBV | No seasonal terms | 3 | 1.0012 (0.9993–1.0031) | +1.4 | 0.216 |
| Antiviral reimbursement HCV | Annual sine/cosine | 0 | 1.0044 (1.0026–1.0061) | +5.4 | <0.001 |
| Antiviral reimbursement HCV | Annual sine/cosine | 1 | 1.0044 (1.0024–1.0063) | +5.4 | <0.001 |
| Antiviral reimbursement HCV | Annual sine/cosine | 6 | 1.0044 (1.0024–1.0063) | +5.4 | <0.001 |
| Antiviral reimbursement HCV | No seasonal terms | 3 | 1.0036 (1.0010–1.0063) | +4.4 | 0.007 |
| Inpatient episodes HBV | Annual sine/cosine | 0 | 1.0074 (1.0020–1.0128) | +9.2 | 0.007 |
| Inpatient episodes HBV | Annual sine/cosine | 1 | 1.0074 (1.0019–1.0128) | +9.2 | 0.008 |
| Inpatient episodes HBV | Annual sine/cosine | 6 | 1.0074 (1.0038–1.0110) | +9.2 | <0.001 |
| Inpatient episodes HBV | No seasonal terms | 3 | 1.0068 (1.0021–1.0115) | +8.4 | 0.005 |
| Inpatient episodes HCV | Annual sine/cosine | 0 | 0.9936 (0.9874–0.9999) | -7.4 | 0.046 |
| Inpatient episodes HCV | Annual sine/cosine | 1 | 0.9936 (0.9876–0.9996) | -7.4 | 0.036 |
| Inpatient episodes HCV | Annual sine/cosine | 6 | 0.9936 (0.9896–0.9977) | -7.4 | 0.002 |
| Inpatient episodes HCV | No seasonal terms | 3 | 0.9950 (0.9911–0.9989) | -5.9 | 0.012 |
| Protocol procedures HBV | Annual sine/cosine | 0 | 1.0100 (1.0022–1.0178) | +12.7 | 0.011 |

| Series | Specification | HAC lag | Monthly ratio and 95% CI | Annualised change % | p |
|---|---|---|---|---|---|
| Protocol procedures HBV | Annual sine/cosine | 1 | 1.0100 (1.0027–1.0173) | +12.7 | 0.007 |
| Protocol procedures HBV | Annual sine/cosine | 6 | 1.0100 (1.0065–1.0136) | +12.7 | <0.001 |
| Protocol procedures HBV | No seasonal terms | 3 | 1.0100 (1.0048–1.0152) | +12.7 | <0.001 |
| Protocol procedures HCV | Annual sine/cosine | 0 | 0.9958 (0.9862–1.0054) | -5.0 | 0.388 |
| Protocol procedures HCV | Annual sine/cosine | 1 | 0.9958 (0.9871–1.0045) | -5.0 | 0.343 |
| Protocol procedures HCV | Annual sine/cosine | 6 | 0.9958 (0.9907–1.0008) | -5.0 | 0.101 |
| Protocol procedures HCV | No seasonal terms | 3 | 0.9980 (0.9899–1.0062) | -2.4 | 0.627 |
| Consultations HBV | Annual sine/cosine | 0 | 1.0030 (1.0002–1.0058) | +3.7 | 0.036 |
| Consultations HBV | Annual sine/cosine | 1 | 1.0030 (1.0003–1.0057) | +3.7 | 0.030 |
| Consultations HBV | Annual sine/cosine | 6 | 1.0030 (1.0015–1.0045) | +3.7 | <0.001 |
| Consultations HBV | No seasonal terms | 3 | 1.0029 (1.0011–1.0046) | +3.5 | 0.001 |
| Consultations HCV | Annual sine/cosine | 0 | 0.9941 (0.9897–0.9985) | -6.9 | 0.009 |
| Consultations HCV | Annual sine/cosine | 1 | 0.9941 (0.9897–0.9985) | -6.9 | 0.009 |
| Consultations HCV | Annual sine/cosine | 6 | 0.9941 (0.9908–0.9974) | -6.9 | <0.001 |
| Consultations HCV | No seasonal terms | 3 | 0.9947 (0.9907–0.9988) | -6.1 | 0.012 |

Model interpretation. With 24 complete service months, only two annual cycles are observed. The models estimate descriptive smooth trends; neither harmonic adjustment nor HAC inference removes uncertainty from the short series or endpoint choice. The complete-year and full-window service trends have the same directions. The HCV protocol trend is compatible with no change, and the magnitude of the expenditure trend, especially for HCV, differs between windows. No causal effect or access outcome is inferred.

Table S21. Observed antiviral identification codes. Canonical ATC codes use each product's most recent observed ATC; historical codes are listed where observed. NHIF codes identify reimbursed products. Drug-identified records additionally require K74.0 or K74.6; a product code alone does not establish infection. This inventory covers the full source window.

| Virus assignment | Canonical ATC | Observed ATC codes | Active substance | Observed NHIF product codes |
|---|---|---|---|---|
| HBV | J05AF05 | J05AF05 | lamivudine | JF417 |
| HBV | J05AF07 | J05AF07 | tenofovir disoproxil | JF464, JF572, JF576, JF579, JF582, JF590, JF593 |
| HBV | J05AF10 | J05AF10 | entecavir | JF462, JF578, JF580, JF581 |
| HBV | J05AF11 | J05AF11 | telbivudine | JF453 |
| HBV | L03AB11 | L03AB11 | peginterferon alfa 2-a | LF079 |
| HCV | J05AB04 | J05AB04 | ribaverin | JF337, JF512 |
| HCV | J05AP51 | J05AP51, J05AX65 | ledipasvir/sofosbuvir | JF569 |
| HCV | J05AP54 | J05AP54, J05AX68 | elbasvir/grazoprevir | JF571 |
| HCV | J05AP55 | J05AP55, J05AX69 | sofosbuvir/velpatasvir | JF575 |
| HCV | J05AP56 | J05AP56 | sofosbuvir, veloatasvir, voxilaprevir | JF585 |
| HCV | J05AP57 | J05AP57 | glecaprevir, pibrentasvir | JF583 |

The HBV diagnosis list is B16, B16.0, B16.1, B16.2, B16.9, B17.0, B18.0, B18.1; the HCV list is B17.1, B18.2. Non-specific codes are B18, B18.8, B18.9, B19, B19.0, B19.9, Z22.5 and are not assigned to HBV or HCV. Peginterferon and ribavirin do not have exclusive indications; their assignments depend on the stated administrative algorithm and context.

Table S22. Case-definition sensitivity during complete years 2021–2025. The main definition combines mutually exclusive diagnosis- and drug-identified records. The diagnosis-only definition excludes all K74.0/K74.6 records; the third definition removes only peginterferon alfa-2a from the drug-identified arm. The table assesses the expenditure consequence of the rules, not diagnostic validity.

| Definition | HBV EUR | HCV EUR | Total EUR | Change from main total % |
|---|---|---|---|---|
| Both arms | 9,960,035.57 | 91,576,404.93 | 101,536,440.50 | +0.000 |
| Diagnosis-identified only | 8,390,788.21 | 72,254,752.72 | 80,645,540.93 | -20.575 |
| Both arms excluding drug-identified peginterferon | 9,938,816.76 | 91,576,404.93 | 101,515,221.69 | -0.021 |

Excluding the entire drug-identified arm reduces observed antiviral reimbursement by 20.575%. Excluding only drug-identified peginterferon reduces the total by 0.021% and HBV reimbursement by 0.213%. Small aggregate impact does not validate the allocation of individual records or the specificity of other medicines.

Table S23. Product-level Fisher decomposition for all complete-year comparisons. Fixed-base rows compare each year with 2021; chained rows compare consecutive years. Factors multiply to the expenditure ratio before rounding. These are the intermediate and chained comparisons not shown in manuscript Table 3.

| Basis | Virus | Comparison | Expenditure ratio | Price index | Quantity index | Entry and exit | Matched products |
|---|---|---|---|---|---|---|---|
| Fixed base | HBV | 2021 to 2022 | 0.993 | 0.971 | 1.034 | 0.989 | 9 |
| Fixed base | HBV | 2021 to 2023 | 0.860 | 0.784 | 1.128 | 0.972 | 8 |
| Fixed base | HBV | 2021 to 2024 | 0.953 | 0.789 | 1.243 | 0.971 | 8 |
| Fixed base | HBV | 2021 to 2025 | 1.083 | 0.799 | 1.395 | 0.971 | 7 |
| Fixed base | HCV | 2021 to 2022 | 0.923 | 0.970 | 0.952 | 0.999 | 5 |
| Fixed base | HCV | 2021 to 2023 | 1.180 | 0.943 | 1.267 | 0.987 | 4 |
| Fixed base | HCV | 2021 to 2024 | 1.169 | 0.901 | 1.313 | 0.987 | 4 |

| Basis | Virus | Comparison | Expenditure ratio | Price index | Quantity index | Entry and exit | Matched products |
|---|---|---|---|---|---|---|---|
| Fixed base | HCV | 2021 to 2025 | 1.157 | 0.885 | 1.447 | 0.903 | 3 |
| Chained | HBV | 2021 to 2022 | 0.993 | 0.971 | 1.034 | 0.989 | 9 |
| Chained | HBV | 2022 to 2023 | 0.866 | 0.806 | 1.079 | 0.996 | 8 |
| Chained | HBV | 2023 to 2024 | 1.108 | 1.006 | 1.101 | 1.000 | 8 |
| Chained | HBV | 2024 to 2025 | 1.136 | 1.012 | 1.123 | 1.000 | 7 |
| Chained | HCV | 2021 to 2022 | 0.923 | 0.970 | 0.952 | 0.999 | 5 |
| Chained | HCV | 2022 to 2023 | 1.278 | 0.972 | 1.318 | 0.998 | 4 |
| Chained | HCV | 2023 to 2024 | 0.991 | 0.956 | 1.036 | 1.000 | 4 |
| Chained | HCV | 2024 to 2025 | 0.990 | 0.980 | 1.011 | 0.999 | 3 |

# References


26. National Health Insurance Fund of Bulgaria. Requirements of the NHIF for the Treatment of Chronic Viral Hepatitis B in Patients over 18 Years of Age in Outpatient Care. Approved by the Manager of the NHIF, Ref. RD-09-633 of 8 July 2025. Available online: https://www.nhif.bg/bg/medical_requirements/definitions (accessed on 26 October 2026).

27. National Health Insurance Fund of Bulgaria. Requirements for Treatment of Chronic Viral Hepatitis C in Adults in Outpatient Care. State Gazette No. 73, Effective 25 August 2023 to 1 September 2025; and No. 72, Effective 2 September 2025 to 16 February 2026. Available online: https://www.nhif.bg/bg/medical_requirements/definitions (accessed on 16 October 2026).

31. von Elm, E.; Altman, D.G.; Egger, M.; Pocock, S.J.; Gøtzsche, P.C.; Vandenbroucke, J.P.; STROBE Initiative. The Strengthening the Reporting of Observational Studies in Epidemiology (STROBE) Statement: Guidelines for Reporting Observational Studies. Lancet 2007, 370, 1453–1457. https://doi.org/10.1016/S0140-6736(07)61602-X.

32. Benchimol, E.I.; Smeeth, L.; Guttmann, A.; Harron, K.; Moher, D.; Petersen, I.; Sørensen, H.T.; von Elm, E.; Langan, S.M.; RECORD Working Committee. The REporting of Studies Conducted Using Observational Routinely-Collected Health Data (RECORD) Statement. PLoS Med. 2015, 12, e1001885. https://doi.org/10.1371/journal.pmed.1001885.

40. National Health Insurance Fund; Bulgarian Medical Association. National Framework Contract No. RD-NS-01-2 of 1 September 2023 for Medical Activities Between the National Health Insurance Fund and the Bulgarian Medical Association for 2023–2025. 2023. Available online: https://www.nhif.bg/ (accessed on 26 October 2026).
